# Molecular characterization and safety properties of multi drug-resistant *Escherichia coli* O157:H7 bacteriophages

**DOI:** 10.1186/s12866-024-03691-w

**Published:** 2024-12-19

**Authors:** Bukola Opeyemi Oluwarinde, Daniel Jesuwenu Ajose, Tesleem Olatunde Abolarinwa, Peter Kotsoana Montso, Henry Akum Njom, Collins Njie Ateba

**Affiliations:** 1 Antimicrobial Resistance and Phage Biocontrol Research Group (AREPHABREG), Department of Microbiology, School of Biological Sciences, Faculty of Natural and Agricultural Sciences, North‒West University, Private Mail Bag X2046, Mmabatho, 2735 South Africa; 2 Food Security and Safety Focus Area, Faculty of Natural and Agricultural Sciences, North‒West University, Private Mail Bag X2046, Mmabatho, 2735 South Africa; 3 Agricultural Research Council, Private Mail Bag X2046, Potchefstroom, 2531 South Africa

**Keywords:** Antibiotic resistance, Biocontrol, *E. coli* O157:H7, Food pathogen, Phage genome, Whole genome sequencing

## Abstract

The increase in multi drug resistance (MDR) amongst food-borne pathogens such as *Escherichia coli* O157:H7, coupled with the upsurge of food-borne infections caused by these pathogens is a major public health concern. Lytic phages have been employed as an alternative to antibiotics for use against food-borne pathogens. However, for effective application, phages should be selectively toxic. Therefore, the objective of this study was to characterise lytic *E. coli* O157:H7 phages isolated from wastewater as possible biocontrol agents and access their genomes for the absence of genes that denotes virulence, resistance, toxins, and lysogeny using whole genome sequencing. *E. coli* O157:H7 bacteriophages showed clear plaques ranging in size from 1.0 mm to 2.0 mm. Spot test and Efficiency of plating (EOP) analysis demonstrated that isolated phages could infect various environmental *E. coli* strains. Four phages; vB_EcoM_EP32a, vB_EcoP_EP32b, vB_EcoM_EP57, and vB_EcoM_EP69 demonstrated broad lytic spectra against *E. coli* O157:H7 strains. Transmission Electron Microscopy (TEM) showed that all phages have tails and were classified as *Caudoviricetes*. Growth parameters showed an average latent period of 15 ± 3.8 min, with a maximum burst size of 392 PFU/cell. The phages were stable at three distinct temperatures (4 °C, 28 °C, and 37 °C) and at pH values of 3.5, 5.0, 7.0, 9.0, and 11.0. Based on their morphological distinctiveness, three phages were included in the Whole Genome Sequencing (WGS) analysis. WGS results revealed that *E. coli* O157:H7 phages (vB_EcoM_EP32a, vB_EcoP_EP32b, and vB_EcoM_EP57) were composed of linear double-stranded DNA (dsDNA) with genome sizes 163,906, 156,698, and 130,723 bp and GC contents of 37.61, 37, and 39% respectively. Phages vB_EcoM_EP32a and vB_EcoP_EP32b genomes were classified under the class *Caudoviricetes*, S*traboviridae* family, and the new genus “*Phapecoctavirus*”, while vB_EcoM_EP57 was classified under the class *Caudoviricetes*, *Autographiviridae* family. Genome analysis revealed no lysogenic (integrase), virulence, or antimicrobial resistance sequences in all three *Escherichia* phage genomes. The overall results provided evidence that lytic *E. coli* O157:H7 bacteriophages in this study, are relatively stable, can infect diverse *E. coli* strains, and does not contain genes responsible for virulence, resistance, toxins, and lysogeny. Thus, they can be considered as biocontrol candidates against MDR pathogenic *E. coli* O157:H7 strains in the food industry.

## Background

Bacteriophages, often known as phages, are naturally occurring organisms that have evolved alongside bacteria for billions of years [1]. They are viruses that target and infect bacteria with great specificity, making them highly efficient as biocontrol agents. They are abundant in nature, with about 4.8 × 10^31^ phage particles existing on the earth [2]. They are ubiquitous and can be isolated in the same environment where their bacteria hosts are found. They are important regulators of bacterial physiology, diversity, abundance, evolution, and pathogenicity in their natural environment [3]. MDR *E. coli* O157:H7 causes food-borne infection and as such become a public health concern, hence phages specific to *E. coli* O157:H7 have now been employed as an alternative therapy for use against food-borne infection [4]. *E. coli* phages play a significant role in both bacterial ecology and potential medical and biotechnological applications. *E. coli* phages possess different characteristics and genetic combinations. Some phages have large genomes with numerous genes, while others are much smaller. While certain phages exhibit lytic behavior, other phages display lysogenic characteristics. These traits are vital when considering the potential use of phages as a biocontrol strategy against *E. coli* O157.

Phage candidates for treating *E. coli* O157:H7 infections should be safe and effective; they should be able to inhibit the disease-causing pathogen without causing any harm to either the host cell, host normal flora, or the environment [5]. Some phages possess virulence and antimicrobial genes, toxins, allergens, and lysogenic properties that are not suitable for biocontrol candidate phages [6]. The presence of these virulence and resistance genes can facilitate their transfer to host bacteria, thereby increasing the pathogenicity of the pathogen. To reduce the possibility of transmitting these genes to bacteria, there is a need to screen phage preparations for the presence of virulence and AMR genes before using them for therapy.

Researchers have suggested strategies to determine phage safety. This includes the use of animal models to monitor allergies and safety; clinical trials in humans have also been conducted in the past to assess the efficiency and safety of phages in humans. However, these methods are quite expensive and have ethical implications and so the use of new generation sequencing techniques like the whole genome sequencing (WGS) can be cost effective and has been a promising tool to analyse and characterise phage genomes to ascertain their suitability as a biocontrol. According to Lee, Choi [7], WGS was used to access the lack of virulence factors, toxins, antibiotic resistance, and allergen-coding genes within the genome of the *E. coli* O157-specific phage KFS-EC and was considered a suitable candidate for biocontrol purposes. Furthermore, Liao, Sun [8] showed that WGS can be employed to identify lysogenic elements that may aid in the spread of unwanted genes to bacterial populations. Hence, this study aimed to isolate and characterize *E. coli* O157:H7 phages and determine the safety of the characterized phages using whole genome sequencing.

## Methods

### Bacteria isolates

Three virulent and MDR *E. coli* O157:H7 (JAVLRS000000000, JAVCZL000000000 and JAURAD000000000) were used as hosts in isolating the phages. From each bacteria isolate, a pure colony was picked and placed into sterile conical flasks containing 15 mL of nutrient broth. The broth culture was incubated at 37 °C with agitation at 180 rpm (Biobase shaking incubator, model: BJPX200N, Shandong, China) until the growth achieved an optical density (OD) ranging from of 0.4 to 0.5 at 600 nm. These competent bacterial cultures were used as host strains for phage isolation.

### Isolation of *Escherichia coli* O157:H7 bacteriophages

*E. coli* O157:H7 bacteriophages were isolated from wastewater samples using the enrichment method [9], with some modifications. One litre of wastewater was obtained from the Mmabatho sewage treatment plant. Samples were obtained from different spots into sterile plastic containers with tight lids. The samples were transported on ice packs to the laboratory for analysis. The Hi Centrifuge SR (Model: Z300, Germany) was used to centrifuge 50 mL of each sample at 10,000 ×g for 10 min to separate particles and other impurities. To get the crude phage filtrates, 10 mL of the supernatant was filtered with a 0.22 μm pore syringe filter (GVS Filter Technology, USA). For the enrichment process, 10 mL of each filtrate was mixed with 10 mL of double-strength tryptic soya broth (TSB) in a 100 mL conical flask. Then, 100 µL of an exponential phase culture of the three *E. coli* O157:H7 (JAVLRS000000000, JAVCZL000000000 and JAURAD000000000) was added to each mixture, and the samples were incubated while shaking (180 rpm) at 37 °C for 24 h. After incubation, the samples were centrifuged at 10,000 ×g for 10 min, the supernatants were then filter-sterilised with 0.22 μm pore-size acrodisc syringe filters (GVS Filter Technology, USA) and used for further experiments.

### Spot test

The spot test was performed using the method described by [10]. Briefly, 100 µL of overnight cultures of a bacteria host in its exponential phase (OD_600_ = 0.4 to 0.5) was mixed with 5 mL of soft agar (0.3% w/v). This mixture was spread-plated onto modified nutrient agar (MNA) plates to make a bacterial lawn. The plates were left at room temperature for 15 min to solidify. The activity of the phage was tested by placing 10 µL of each lysate on top of the bacterial lawn on soft agar and left to dry for 10 min. The plates were then incubated at 37 °C for 24 h. After incubation, the plates were observed for the presence of plaques along the sites of inoculation. Visible clear plaques were carefully cut out from the overlays (soft-agar layers) with a 1000 µL pipette tip and put into 300 µL of lambda diluent (10 mM Tris Cl (pH 7.5), 8 mM MgSO_4_.7H_2_O) contained in a 2 mL Eppendorf tube and this was done for each plaque. The tubes were left out at room temperature overnight so that phage particles could diffuse through the soft agar into the buffer. This mixture was then centrifuged at 10,000 rpm for 10 min, and the supernatant was filtered through 0.22 μm Millipore membrane syringe filters (Fischer Scientific, Ottawa, ON). The filtered phage lysates were used for the purification process.

### Phage purification

Phages were purified using the plaque assay as previously described [10]. Briefly, ten-fold serial dilutions of crude phage lysates were prepared, and an aliquot of 100 µL of each phage lysate was mixed with 100 µL of exponential phase (OD_600_ = 0.4 to 0.5) culture of the corresponding host(s). The tubes were left for 10 min at room temperature to allow the phage to attach to the host. The contents of the tube were mixed with 3 mL of soft agar (0.3% w/v), then poured onto MNA plates. The plates were left to solidify and then incubated at 37 °C for 24 h. After incubation, the plates were examined for distinct plaques. Sterile pipette tips were used to select plaques based on size and clarity from each plate and resuspended in 1 mL lambda diluent in 2 mL Eppendorf tubes. Phage was allowed to diffuse into the solution for 24 h and then centrifuged at 10,000 rpm for 10 min. The supernatant was filter-sterilised using a 0.22 μm pore size filter. The purification process was done three times to obtain uniform plaques.

### Propagation and titration of *E. coli* O157:H7 bacteriophages

*E. coli* O157:H7 isolates were used to propagate purified phages. A 100 µL aliquot of pure phage stocks were added to 100 µL of bacteria hosts at exponential phase (OD_600_ = 0.4 to 0.5) in 100 mL conical flasks containing 10 mL of sterile double-strength TSB. Each conical flask was incubated at 37 °C for 24 h in a shaking incubator (model: BJPX200N, Shandong, China) at 180 rpm. After incubation, the samples were centrifuged at 10,000 ×g for 10 min at 4 °C, and the supernatant was filtered using an acrodisc syringe filter with a 0.22 μm pore size. Ten-fold serial dilutions were prepared, and plaque assay was carried out to obtain phage titres. An aliquot of 100 µL of each phage lysate was serially diluted 10-folds and the lysate was mixed with 100 µL of the bacterial host suspensions in test tubes, followed by addition of 3 ml of 0.3% (w/v) top agar. The contents of the tubes were immediately poured onto solidified MNA plates, allowed to solidify for 15 min and the plates were incubated at 37 °C for 24 h. The experiment was carried out in triplicate and results were expressed as plaque-forming units per millilitre (PFU/mL) of lysate. The stock phages were kept at 4 °C for subsequent experiments.

### Determination of host range and cross infectivity of the phage isolates

A total of seventeen phage isolates were obtained after purification. However, based on their uniqueness and formation of clear plaques on spot testing (full lysis), eight phage isolates were selected and tested against 25 bacterial hosts. These included 16 environmental strains (8 *E. coli*, 4 *Staphylococcu*s sp., 1 *Brevibacterium* sp., 1 *Salmonella typhi*, 1 *Campylobacter* sp., and 1 *Vibrio cholerae*) and 9 control strains *(Klebsiella pneumonia* 1388, *E. coli* ATCC 25922, *Pseudomonas aeruginosa* ATCC 27853, *Bacillus cereus* ATCC 10876, *S. aureus* 43300, *S. aureus* ATCC 25923, *K. pneumoniae* ATCC BAA 1075, and *P. aeruginosa* ATCC 43170). These host strains include both Gram-positive and Gram-negative bacteria to broaden the host range. The spot test was used to determine phage lytic spectrum of activity [11]. Briefly, 100 µL of an overnight culture of a bacterial host in its exponential phase (OD_600_ = 0.4 to 0.5) was mixed with 3 mL of soft agar (0.3% w/v). This mixture was then spread onto modified nutrient agar (MNA) plates to prepare a bacteria lawn. The plates were left for 15 min to solidify. The activity of the phages were tested by placing 10 µL of each lysate (10^8^ to 10^9^ PFU/mL) on the bacterial lawn on soft agar and were left for 10 min to dry. After this, the plates were incubated at 37 °C for 24 h. Plaques observed at the point of application were characterised based on their clarity (clear, turbid, and no lysis) [12]. Three independent experiments were carried out to ascertain the validity of the results.

### Efficiency of plating (EOP) of phages

Following the host range experiment and using the presence of lysis as basis, 9 bacteria (7 *E. coli* strains, 1 environmental *S. aureus*, and 1 *P. aeruginosa ATCC 27853)* strains were selected for the “efficiency of plating” (EOP) experiment. The EOP experiment was done to determine the lytic efficiency of phages on other bacteria different from their suitable host bacteria [13]. The suitable host bacteria (*E. coli* O157:H7 -J32, J57, and J69) were used as references. Plaque assay was then carried out as described by Sambrook and Russell [10]. Briefly, ten-fold serial dilutions of phage lysates were prepared, and an aliquot of 100 µL of each phage lysate (1 × 10^6^ PFU/mL) was mixed with 100 µL of exponential phase (OD_600_ = 0.4 to 0.5) bacteria culture. The tubes were left for 10 min at room temperature to allow the phage to attach to the host. The contents of the tube were mixed with 3 mL of soft agar (0.3% w/v), then poured onto MNA plates. For each phage isolate, the experiment was carried out in triplicate. The plates were left to solidify and then incubated at 37 °C for 24 h. The number of plaques formed was counted and relative EOP was determined using the formula below.

$$\:\text{R}\text{e}\text{l}\text{a}\text{t}\text{i}\text{v}\text{e}\:\text{E}\text{O}\text{P}=\frac{\text{a}\text{v}\text{e}\text{r}\text{a}\text{g}\text{e}\:\text{n}\text{u}\text{m}\text{b}\text{e}\text{r}\:\text{o}\text{f}\:\text{p}\text{l}\text{a}\text{q}\text{u}\text{e}\text{s}\:\text{o}\text{n}\:\text{t}\text{a}\text{r}\text{g}\text{e}\text{t}\text{e}\text{d}\:\text{h}\text{o}\text{s}\text{t}\:\text{b}\text{a}\text{c}\text{t}\text{e}\text{r}\text{i}\text{a}}{\text{a}\text{v}\text{e}\text{r}\text{a}\text{g}\text{e}\:\text{n}\text{u}\text{m}\text{b}\text{e}\text{r}\:\text{o}\text{f}\:\text{p}\text{l}\text{a}\text{q}\text{u}\text{e}\text{s}\:\text{o}\text{n}\:\text{r}\text{e}\text{f}\text{e}\text{r}\text{e}\text{n}\text{c}\text{e}\:\text{h}\text{o}\text{s}\text{t}\:\text{b}\text{a}\text{c}\text{t}\text{e}\text{r}\text{i}\text{a}}$$

EOP values were classified as high (EOP ≥ 0.5), i.e., when the productive infection on the target bacterium resulted in at least 50% of the PFU found for the primary host, moderate (0.1 < 0.5), and low (EOP ≤ 0.1) based on the reproducible infection on the targeted bacteria. An EOP ≤ = 0.001 was classified as inefficient [14].

### Polyethylene glycol precipitation assay

Polyethylene glycol (PEG) precipitation (10% w/v) was carried out on phage samples following the method of Gill and Hyman [15]. Phages were propagated to obtain high titers (10^8^ to 10^11^ PFU/mL), after which 40 mL of each phage sample was concentrated in 50 mL falcon tubes by adding 10% (w/v) PEG, and the mixture was incubated overnight at 4 °C to allow the phage particles to precipitate. The next day, phage particles were separated by centrifugation at 4,200 g for 10 min at 4 °C. The supernatant was discarded, and the pellet was washed three times with 1 mM NaNO_3_. The pellet was then reconstituted in 200 µL of ammonium acetate. The samples were packed in a cooler box containing ice packs and transported to the Virology Laboratory at the North-West University, Potchefstroom Campus, for transmission electron microscopy (TEM) analysis.

### Transmission electron microscopy (TEM) analysis

To investigate the morphology of the phages, the TEM technique [16] was used. Preparation of the samples and TEM was done at the Electron Microscopy lab, Chemical Resource Beneficiation, Building G10, Room G24, Potchefstroom Campus, North-West University, Potchefstroom, South Africa. Four phage isolates were subjected to TEM, and the phage morphotype was determined using negative staining techniques as previously described [17], with some modifications. A drop of concentrated phage solution was deposited on 300 mesh copper grids with carbon-support films. The phage particles were allowed to adsorb for 2 min, the excess liquid was drained off with a filter paper and the grid was allowed to dry. The suspension was stained with 1% (w/v) aqueous ammonium molybdate (pH = 7.0) for 5 min, the excess fluid was drawn off with filter paper and allowed to air dry for 5 min. The grid containing the specimen (phage particles) was examined with a 200 kV FEG FEI Tecnai G2 F20 TEM equipment that is designed for high-end conventional and high-resolution analysis at a magnification range of 15,000–20000X. Micrographs were taken with a Gatan bottom-mount camera and Digital Micrograph software. The sizes of phage structures were evaluated by measuring 10 particles of each phage and calculating the average,

### Determination of phage growth parameters

The life cycle of phage isolates was studied using a modified version of the one-step growth approach that was previously described [11]. Briefly, 5 mL of each host’s exponential phase (10^7^ CFU/mL) culture was centrifuged at 8,000 g for 5 min at 4 °C. The pellet was then resuspended in 10 mL of double-strength TSB supplemented with CaCl_2_ to achieve an OD between 0.40 and 0.50 at 600 nm. The bacterial concentration was determined by diluting an aliquot of 100 µL in a ten fold serial dilution and plating on nutrient agar. One hundred microliters of each phage isolate (10^8^ PFU/mL) were added to their respective host bacteria solution at a multiplicity of infection (MOI) of 1. The mixture was left at room temperature for 10 min for phage-host attachment. The mixture was then centrifuged at 11,000 xg for 10 min to remove phage particles that had not been adsorbed. The pellet was resuspended in 0.1 mL of TSB and then transferred to 9.9 mL of pre-warmed TSB. The samples were incubated in a shaking incubator (180 rpm) at 37 °C for one hour. Every 5 min, 100 µL of the mixture was withdrawn from each sample, and then a plaque assay was carried out in triplicate to evaluate the phage titer. The results obtained were used to calculate the latent period, burst time, and phage relative burst size for each infected cell. The relative burst size was estimated following the formula of El-Dougdoug, Cucic [18]:

$$\:\text{R}\text{e}\text{l}\text{a}\text{t}\text{i}\text{v}\text{e}\:\text{b}\text{u}\text{r}\text{s}\text{t}\:\text{s}\text{i}\text{z}\text{e}=\frac{\text{f}\text{i}\text{n}\text{a}\text{l}\:\text{t}\text{i}\text{t}\text{e}\text{r}\:\left(\frac{\text{p}\text{f}\text{u}}{\text{m}\text{L}}\right)-\text{i}\text{n}\text{i}\text{t}\text{i}\text{a}\text{l}\:\text{t}\text{i}\text{t}\text{e}\text{r}\:(\text{p}\text{f}\text{u}/\text{m}\text{L})}{\text{f}\text{i}\text{n}\text{a}\text{l}\:\text{t}\text{i}\text{t}\text{e}\text{r}\:(\text{p}\text{f}\text{u}/\text{m}\text{L})}$$

The relative burst size was presented in Log scale (Log _10_) and plotted against time to determine the latent period and burst size of each phage isolate.

### Adsorption test

An adsorption test was carried out to determine the rate at which phages adsorb to the host bacteria following the method described by Vukotic, Obradovic [19], with a slight modification. An aliquot (100 µL) of phage lysate (10^8^ PFU/mL) was added to 100 µL of exponential phase bacterial culture (10^7^ CFU/mL), after which 100 µL of the mixture was added to 900 µL of the phage buffer. This was done at time intervals of 1, 3, 5, 10, and 15 min. This was then centrifuged for 5 min at 11,000 g. The supernatant was then transferred into a sterile tube and used in a plaque assay to determine the concentration of unadsorbed phage. The experiment was carried out in triplicate. Percentage of adsorbed phages was calculated by the formula as described by Vukotic, Obradovic [19].

$$\:\text{A}\text{d}\text{s}\text{o}\text{r}\text{b}\text{e}\text{d}\:\text{p}\text{h}\text{a}\text{g}\text{e}\text{s}\left(\%\right)=1-\frac{\text{t}\text{i}\text{t}\text{r}\text{e}\:\text{o}\text{f}\:\text{s}\text{u}\text{p}\text{e}\text{r}\text{n}\text{a}\text{t}\text{a}\text{n}\text{t}}{\text{S}\text{t}\text{o}\text{c}\text{k}\:\text{t}\text{i}\text{t}\text{r}\text{e}}\times\:100$$

### Effect of temperatures on the stability and viability of phages

Firstly, it is noteworthy to state that cooling temperature (4 °C), room temperature (28 °C) and body temperature (37 °C) are significant in the food industry. The effect of temperature was evaluated across these temperatures (4, 28, and 37 °C) in a temperature-controlled incubator. The concentrations of the host bacteria and phage titers were standardised before starting the experiment. One hundred microliters of exponential phase culture (10^5^ CFU/mL) were added to 100 µL of phage (10^5^ PFU/mL) and left to stand at 4, 28, and 37 °C for 15 min, after which the plaque assay was done in triplicate. Simultaneously, phage stability was evaluated by exposing phage lysates at 40 °C for 60 min, after which 100 µL of phage was pipetted at 15, 30, and 60 min and added to 100 µL of exponential phase bacteria culture. Viability and stability at the different temperatures were carried out using double-layer agar [10]. Plaque assays were performed in triplicate for each sample, and the results were expressed as PFU/mL.

### Effect of different pH levels on the stability and viability of phages

The stability and viability of phages were assessed at various pH values (3.5, 5.0, 7.0, 9.0, and 11.0). This was done during a 24- to 48-hour incubation period. TSB medium was adjusted with 1 M hydrochloric acid (HCL) or sodium hydroxide (NaOH) to obtain the varying pH studied. Ten millilitres of adjusted sterile double-strength TSB were distributed into 50 mL falcon tubes. One hundred microliters of each bacterial host (10^5^ CFU/mL) and their corresponding phage (10^5^ PFU/mL) isolates were added to the flask. The tubes were incubated in a shaking incubator (180 rpm) at 37 °C for 24 h. A spot test was done after 24 and 48 h to determine the viability of the phage.

To determine the stability of phage lysates, aliquots of 900 µL of pH-adjusted TSB were transferred into Eppendorf tubes and labelled accordingly, after which 100 µL of phage lysates were added to each tube and mixed properly. The tubes were allowed to stand at room temperature. Samples were collected at 24 and 48 h, and phage titre was measured for each sample using a standard plaque assay [10]. Plaque assay was done in triplicate, and the results were represented as PFU/mL.

### Phage DNA extraction

Due to their morphological distinctiveness, DNA was extracted from three phages (*Escherichia* phage vB_EcoM_EP32a, vB_EcoP_EP32b, and vB_EcoM_EP57) and were characterized molecularly. Phage DNA extraction was performed using the phenol-chloroform-isoamyl alcohol method, as previously outlined by Zhao, Sun [20], with some modifications. Initially, the phage stock was treated with 10% (w/v) PEG 8000 and then incubated at 4 °C for 24 h for phage particle precipitation. The resulting mixture was subjected to centrifugation at 10,000 × g for 10 min at 4 °C. The phage pellet was washed three times with 1 mL of lambda diluent (containing 5.8 g/L NaCl, 2 g/L MgSO4·7H2O, and 10 mL/L Tris-HCl (pH 7.5)) to eliminate residual PEG. Subsequently, the pellet was resuspended in 500 µL of lambda diluent. After which 18 µL of DNase I (0.8 U/mL) and 8 µL of RNase (0.1 mg/mL) were added to the sample for residual DNA or RNA degradation. The mixture was then incubated for 30 min at 37 °C. After incubation, 50 µL of 20% (w/v) sodium dodecyl sulphate (SDS) and 18 µL of 10 mg/mL proteinase K were introduced into the sample. The mixture was carefully inverted three times and incubated for an additional 30 min at 37 °C. Subsequently, 500 µL aliquots were transferred into a new 1.5 µL Eppendorf tube. To each tube, an equal volume (500 µL) of phenol-chloroform-isoamyl alcohol (25:24:1, v/v/v) was added. After five careful inversions, the tubes were centrifuged at 10,000 × g for 5 min at 4 °C. The aqueous layer was carefully pipetted into new 1.5 µL Eppendorf tubes, and the phenol-chloroform-isoamyl alcohol step was repeated. An aliquot of 500 µL of chloroform and isoamyl alcohol (24:1, v/v) was then added and centrifuged at 10,000 × g for 5 min at 4 °C. The aqueous layer was then transferred to a new Eppendorf tube. For the precipitation process, 45 µL of 3 M sodium acetate (pH 5.2) and 500 µL of absolute isopropanol were added to the tube containing phage DNA. The mixture was incubated at -20 °C overnight to allow phage DNA precipitation. The following day, the mixture was centrifuged at 14,800 g for 20 min. The resulting pellet was washed twice with 500 µL of 70% ice-cold ethanol and was subsequently resuspended in 30 µL of TE buffer (10 mM Tris-HCL, 1 mM EDTA, pH 8.0).

### Phage DNA purification and quantification

Phage genomic DNA was purified using Norgen phage DNA isolation kit (Norgen Bioteck Corp., Ontario Canada), following the manufacturer’s instructions. DNA quantity was assessed using NanodropTM -Lite Spectrophotometer (Thermo Fisher Scientific Limited).

### Whole genome sequence of Escherichia phage vB_EcoM_EP32a, vB_EcoP_EP32b, and vB_EcoM_EP57

#### Library preparation and sequencing

Three phages (vB_EcoM_EP32a, vB_EcoP_EP32b, and vB_EcoM_EP57) were selected for whole genome analysis. Phage DNA was sequenced at Inqaba Biotechnical Industry (Pty) (Pretoria, South Africa), a commercial next generation sequence (NGS) service provider. Briefly, samples were fragmented using an enzymatic approach (NEBNext Ultra II FS kit) (https://international.neb.com/products/e7805-nebnext-ultra-ii-fs-dna-library-prep-kit-for-illumina#Product%20Information). Resulting DNA fragments were size selected (> 200 bp), using AMPure XP beads, the fragments were end repaired and Illumina specific adapter sequences were ligated to each fragment. Each sample was individually indexed, and a second size selection step was performed. Samples were then quantified, using a fluorometric method, diluted to a standard concentration (4nM) and then sequenced on Illumina’s NextSeq500 platform, using a NextSeq (300 cycle) kit, following a standard protocol as described by the manufacturer. For each sample, 100 Mb of data (2 × 150 bp long paired-end reads) were produced for each sample. The quality of the raw reads was assessed by FastQC (www.bioinformatics.babraham.ac.uk/projects/fastqc/) [21]. The quality reads were filtered, and the barcodes used were trimmed using Trimmomatic (with parameters set at default) [22]. *De novo* assembly of the trimmed reads was carried out using Spades version 3.13.0.

#### Bioinformatics analysis and annotation of Escherichia phages

The genomes of *Escherichia* phages vB_EcoM_EP32a, vB_EcoP_EP32b, and vB_EcoM_EP57 were subjected to annotation using several online tools. These tools included the Rapid Annotation Using Subsystem Technology (RAST) server (version 2.0), the Pathosystems Resource Integration Centre (PATRIC, version 3.5.43), and the Phage Search Tool (PHAST). Virulence and antibiotic resistance genes within these genomes were assessed using VirulenceFinder version 2.0 and ResFinder version 2.2, respectively, on the Centre for Genomic Epidemiology (CGE) online platform, with a 95% identity threshold [23, 24], and confirmed using PhageLeads on phageleads.dk [25]. Open reading frames (ORFs) within the genomes were predicted using ORFFinder with default parameters (www.ncbi.nlm.nih.gov/orffinder/). The putative functions of these ORFs were confirmed using BLASTp (https://blast.ncbi.nlm.nih.gov/Blast.cgi). Additionally, a search for phage-encoded tRNA genes was conducted using the tRANscan-SE programme, with default parameters (http://lowelab.ucsc.edu/cgi-bin/tRNAscan-SE2.cgi). A circular genome map was generated using Proksee (https://proksee.ca/). The GC content and GC skew of the three *Escherichia* phage genomes were determined using Proksee (https://proksee.ca/). Predicted proteins in the genome were annotated using BLASTp against the NCBI non-redundant GenBank database, and similar amino acid proteins were obtained from Genbank and used in generating a phylogenetic tree using MEGA 11 software.

#### Phylogenetic analysis and genome comparison

A phylogenetic analysis of all phage genomes was conducted to determine the identity similarities between the *Escherichia* phage genomes (vB_EcoM_EP32a, vB_EcoP_EP32b, and vB_EcoM_EP57) and other phage genomes present in the NCBI database. Assembled genomes of all three phages were uploaded to the Virus Classification and Tree Building Online Resource (Viptree) [26] using default settings, dsDNA nucleic acid type, and prokaryote host categories. Thirty-one phage genomes with identity similarities ranging from 97.53 to 99.16% and query coverage ranging from 91 to 95% were selected and used in drawing the phylogenetic tree. A total of 16 phage genomes were selected (closest taxa) from the resultant ViPTree analysis and were used along with the test phage isolates to generate a taxonomic classification using VIRIDIC platform. A hit map showing genetic similarities between the genomes was generated. Easyfig 2.1 visualisation tool was further used to create a diagram depicting the comparative analysis of phage genomes with their close homologs in BLASTn [27].

## Results

### Isolation, purification, and propagation of bacteriophages

**Fig. 1:**
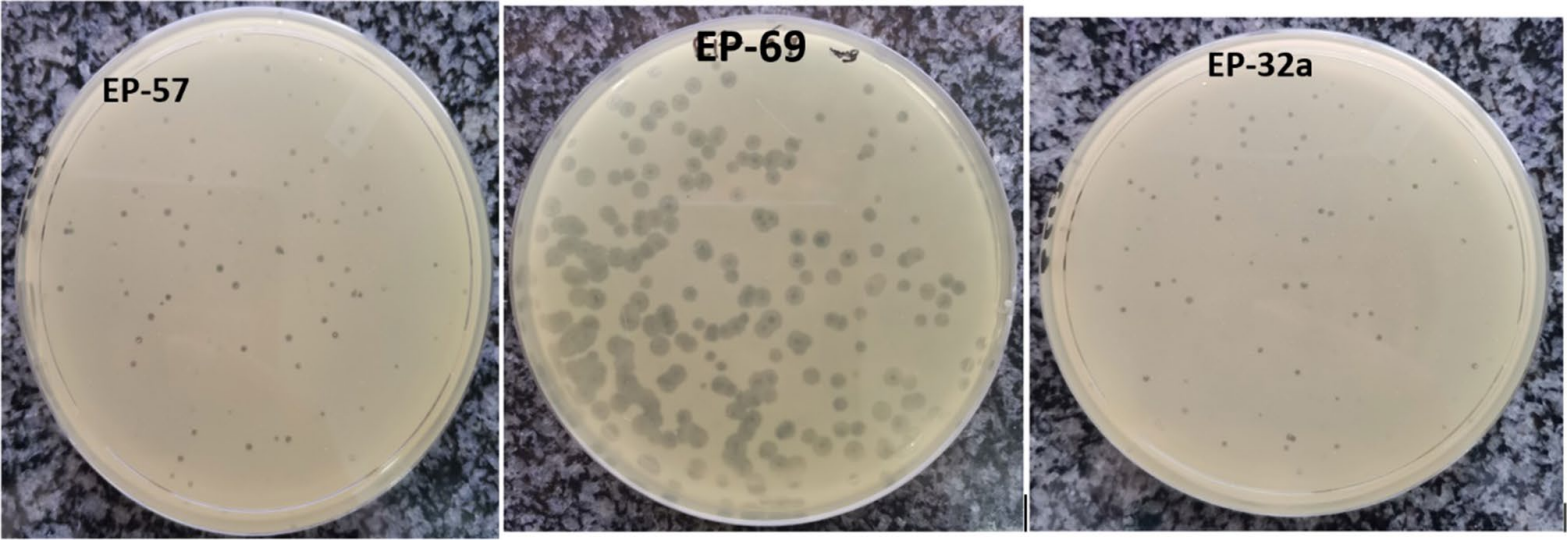
Plaque morphology of representative phage

Twenty sewage samples were obtained and tested for the presence of bacteriophages. From these, three different plaque types were observed on lawns of *E. coli* O157:H7. Phage isolates revealed different plaque morphology in terms of sizes, ranging from small (1 mm) to large (2 mm) plaques. The plaque type and sizes of the phage isolates are shown in Fig. 1. Phage titer after propagation ranged from 14 × 10^5^ to 35 × 10^9^ PFU/mL. From the 20 sewage samples, a total of 17 phage isolates were able to infect the three *E. coli* O157:H7 hosts (JAVLRS000000000, JAVCZL000000000 and JAURAD000000000), and these were used for further analysis.

### Host range and EOP analysis of phages against different *Escherichia coli* strains

The spot test was used to determine the host range of the phages. The results indicate that the phages could infect the environmental *E. coli* species (*E. coli* O157 – J57, J32, J69, J54, J25, J50, J37) while some of the phages were able to infect *Staphylococcus aureus* and *Pseudomonas aeruginosa* ATCC 27,853. None of the phages could infect the other tested bacterial strains (*B. cereus* ATCC 10876, and *K. pneumoniae* ATCC BAA 10705), (Table 1).

**Table 1:** Host range of *E. coli* O157:H7 phages

Bacteria host	Phages							
	EP32a	EP32b	EP57	EP57a	EP57b	EP69	EP69a	EP69b
*E. coli* O157 – J57^a^	+	+	+	+	+	+	+	-
*E. coli* O157 – J32^a^	+	+	+	-	+	+	+	-
*E. coli* O157 – J69^a^	+	+	+	-	+	+	+	+
*E. coli* O157 – J54^a^	+	+	+	+	-	+	-	+
*E. coli* O157 – J25^a^	+	+	+	+	+	+	+	-
*E. coli* O157 – J50^a^	+	+	+	+	-	+	+	+
*E. coli* O157 – J37^a^	+	+	+	+	+	+	+	+
*E. coli* O177^b^	-	-	+	-	-	-	-	-
*S. typhi* ^a^	-	-	-	-	-	-	-	-
*Campylobacter sp.* ^a^	-	-	-	-	-	-	-	-
*V. cholera* ^a^	-	-	-	-	-	+	-	-
*Staphylococcus species M* ^a^	-	-	-	-	-	-	-	-
*Brevibacterium species* ^a^	+	-	-	-	-	-	-	-
*Staphylococcus species S* ^a^	-	-	-	-	-	-	-	-
*Staphylococcus species T* ^a^	+	-	-	-	-	-	-	-
*Staphylococcus aureus U* ^a^	-	-	+	+	+	+	+	+
*K. pneumonia 13,883* ^b^	-	-	-	-	-	-	-	-
*P. aeruginosa ATCC 27,853* ^b^	-	-	+	+	+	-	+	+
*B. aureus ATCC 10,876* ^b^	-	-	-	-	-	-	-	-
*S. aurues 43,300* ^b^	-	-	-	-	-	-	-	-
*S. aureus 2693* ^b^	-	-	-	-	-	-	-	-
*P. multocida* ^b^	-	-	-	-	-	-	-	-
*E. coli 25,922* ^b^	-	-	-	-	-	-	-	-
*K. pneumoniae ATCC BAA 10,705* ^b^	-	-	-	-	-	-	-	-
*P. aeruginosa 48,170* ^b^	-	-	-	-	-	-	-	-

Key: Superscripts “a and b” denotes environmental and control strains respectively, + = Lysis; - = No plaques

The EOP analysis was performed on nine (7 *E. coli* strains, 1 environmental *S. aureus*, and 1 *P. aeruginosa* (ATCC 27853) bacterial isolates that were susceptible to phages on the spot tests. Although the spot test results revealed that these isolates formed clear plaques, the EOP results exhibited various lytic patterns for the phages. Results showed high EOP analysis (EOP ≥ 0.5) on *E. coli* strains and low EOP (EOP ≤ 0.1) on *S. aureus* and *P*. *aeruginosa* (Table 2). Only phage EP32a showed high EOP for all *E. coli* isolates tested, while the other 7 phages showed moderate EOP values (EOP > 0.1 < 0.5) for *E. coli* isolates tested. Generally, EOP values ranged from 0.1 to 0.8. Four phages (EP32a, EP32b, EP57, and EP69) with the greatest broad spectrum of activity were selected for further characterization.

**Table 2:** Efficacy of plating (EOP) of phages against different bacteria host

Bacteria host	Phages							
	EP32a	EP32b	EP57	EP57a	EP57b	EP69	EP69a	EP69b
*E. coli* O157:H7 - J57*	0.7	0.6	1	1	1	0.5	0.7	0.6
*E. coli* O157:H7 - J32*	1	1	0.2	0.6	0.2	0.7	0.7	0.8
*E. coli* O157:H7 - J69*	0.6	0.3	0.5	0.5	0.1	1	1	1
*E. coli* O157:H7 - J54	0.7	0.5	0.1	0.4	0.5	0.2	0.3	0.4
*E. coli* O157:H7 - J25	0.5	0.3	0.5	0.5	0.6	0.4	0.5	0.3
*E. coli* O157:H7 - J50	0.6	0.4	0.6	0.7	0.4	0.8	0.3	0.5
*E. coli* O157:H7 - J37	0.5	0.6	0.5	0.4	0.5	0.6	0.7	0.5
*S. aureus*	-	-	0.1	0.1	0.1	0.2	0.2	0.2
*P. aeruginosa* ATCC 27,853	-	-	0.1	0.04	0.1	0.2	0.1	-

Key: * = reference phages

### Morphological characterisation of phages based on transmission electron microscopy

**Fig. 2:**
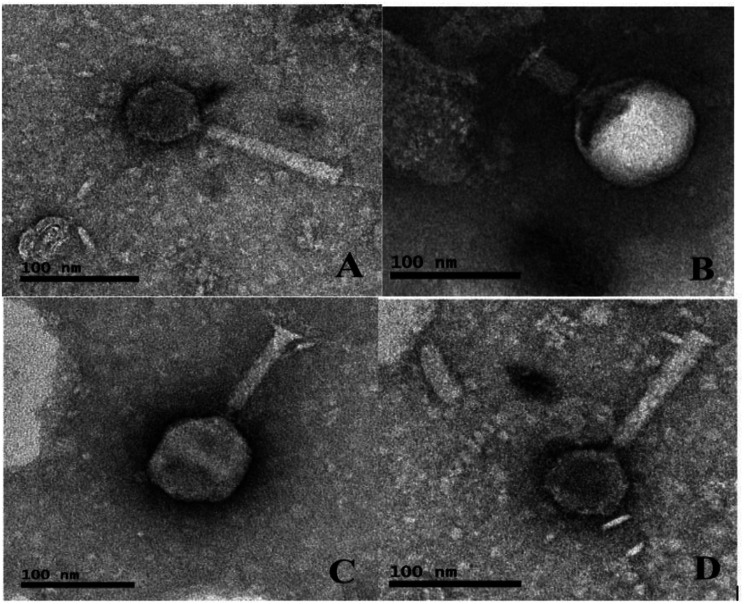
Transmission electron micrographs of representative phage isolates (negatively stained with 1% ammonium molybdate). The bars indicate scale (100 nm). *A = vB_EcoM_EP57; B = vB_EcoP_EP32b; C = vB_EcoM_EP32a and D = vB_EcoM_EP69

Four selected phages chosen from the EOP analysis were subjected to TEM analysis to determine their morphotypes. Transmission electron micrographs of the phages and structural dimensions are shown in Fig. 2; Table 3, respectively. Despite their varying morphotypes on TEM analysis, all the phages belong to the class *Caudoviricetes* due to the possession of a tail. This classification was done according to the ICTV classification for tailed phages. Phage vB_EcoM_EP57 had the longest non-contractile tail of 125 nm with fibres, while phage vB_EcoP_EP32b had the shortest tail of 60 nm.

**Table 3:** Phage dimensions based on TEM analysis

Phage ID	Head structure	Head dimensions (nm)	Tail structure	Tail dimensions (nm)
vB_EcoM_EP32a	Icosahedral head	80	Contractile tail	96
vB_EcoP_EP32b	Icosahedral head	100	Short tail	60
vB_EcoM_EP57	Icosahedral head	60	Contractile tail	125
vB_EcoM_EP69	Icosahedral head	60	Contractile tail	120

### Bacteriophage growth parameters

**Fig. 3:**
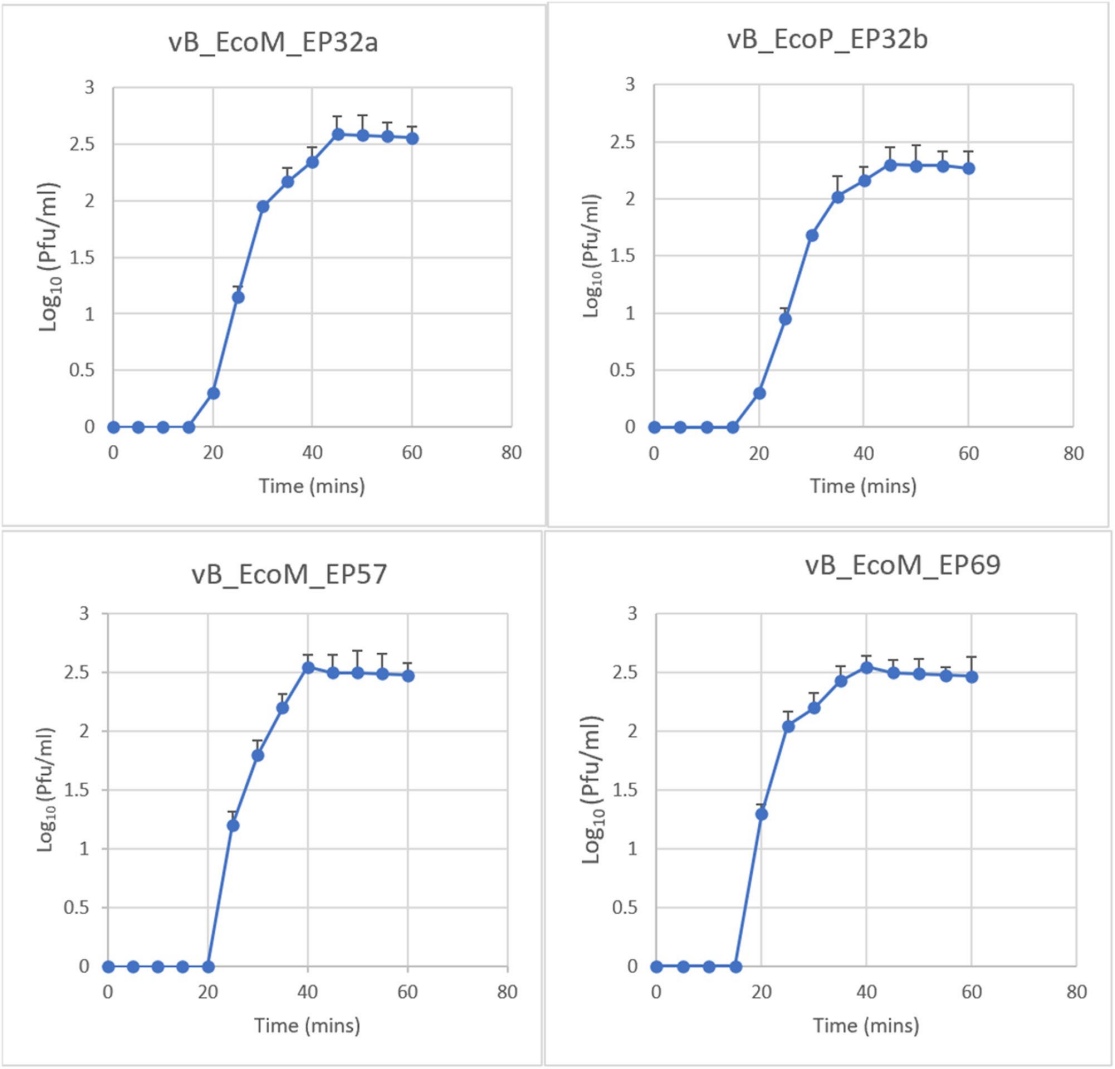
One-step growth curves for four *E. coli* O157:H7 phage isolates

Growth parameters for the four phage isolates were determined using the one-step growth curve. The latent period and relative bust size were determined per infected bacteria cell (Fig. 3). The latent periods for all the phages ranged from 15 to 20 min (average = 15 ± 3.8 min). Phage vB_EcoM_EP57 had the longest latent period of 20 min, while phages vB_EcoM_EP32a, vB_EcoP_EP32b, and vB_EcoM_EP-69 had latent periods of 15 min. In terms of burst size, phages vB_EcoM_EP32a and vB_EcoM_EP69 had the largest burst sizes per infected cell amounting to 392 PFUs and 360 PFUs, respectively, while vB_EcoP_EP32b had the smallest burst size of 200 PFUs per infected cell.

### Adsorption rate of *Escherichia coli* O157:H7 phage

**Fig. 4:**
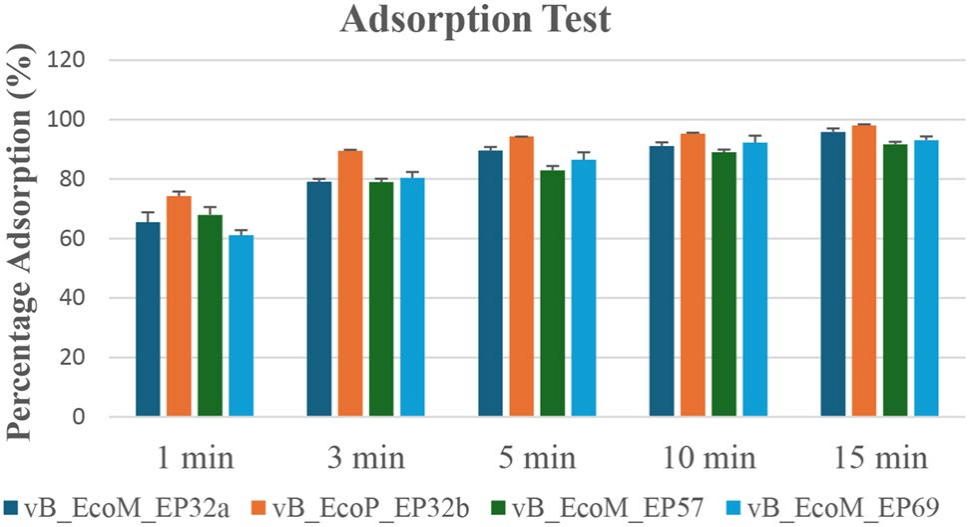
Adsorption rate of *E. coli* O157:H7 phages

The adsorption rates of all four phages are represented in Fig. 4. After 1 min of phage-bacteria incubation, an average of 67.22 ± 5.5% of all phage particles were attached to the host cells, and after 15 min, more than 95% of vB_EcoM_EP32a and vB_EcoP_EP32b were adsorbed, whereas vB_EcoM_EP57 and vB_EcoM_EP69 had adsorption rates of 91.65% and 93.13% after 15 min, respectively.

### Effect of temperature on *E. coli* O157:H7 phages

**Fig. 5:**
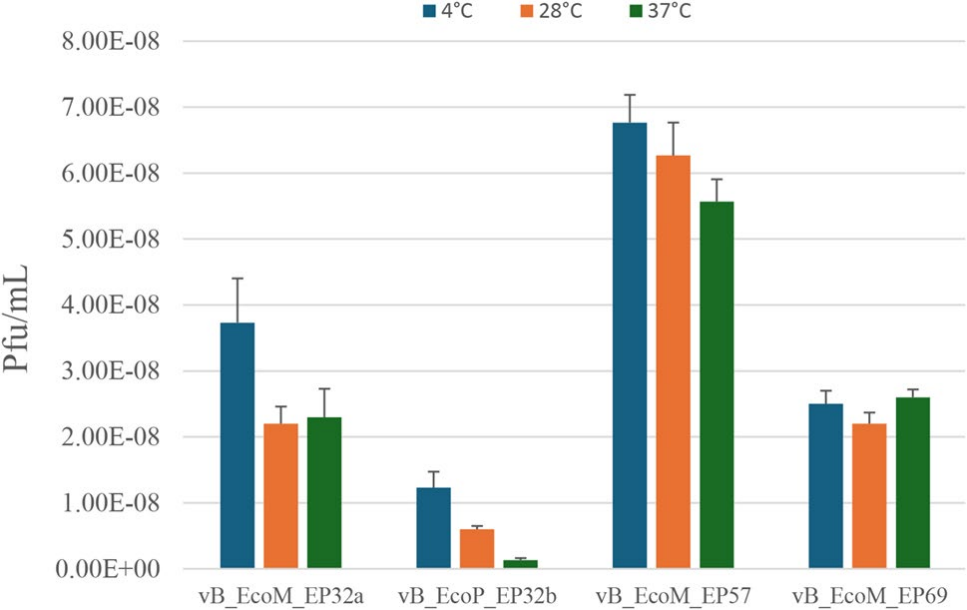
Effect of temperature on *E. coli* O157:H7 phages

The effect of temperature on the phages showed varying results for all four phage candidates (Fig. 5). Results showed that all four phages were active at the studied temperatures. Phage vB_EcoM_EP69 had an almost similar activity at 4 ºC and 37 ºC with concentrations of 25 × 10^8^ pfu/ml and 26 × 10^8^ pfu/ml, respectively. There was a reduction in phage concentration at 28 ºC and 37 ºC for phages vB_EcoM_EP32a, vB_EcoP_EP32b, and vB_EcoM_EP57.

**Fig. 6:**
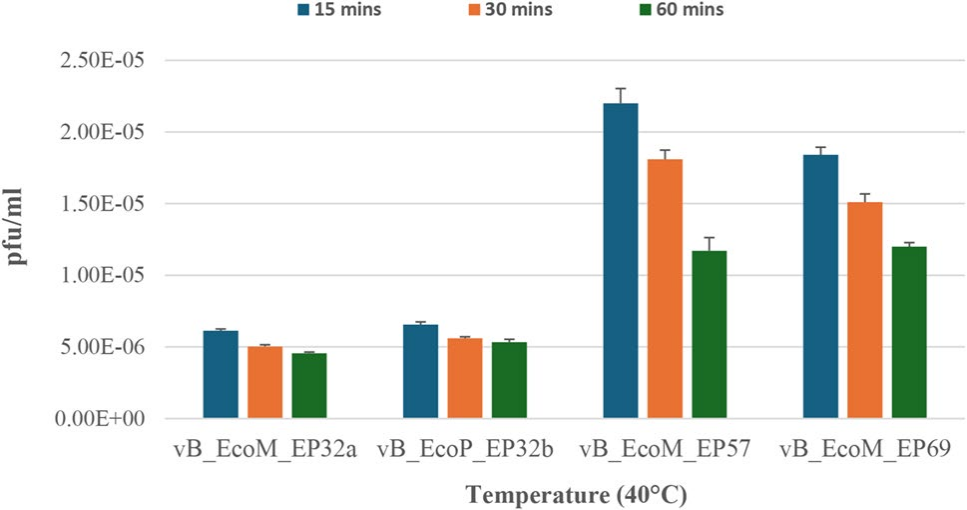
The stability and viability of *E. coli* O157:H7 phage at 40 ºC

The stability and viability of the phages at a high temperature of 40 ºC at different time intervals were also assessed. Incubation of phages for 15 to 60 min resulted in reduced phage growth for vB_EcoM_EP32a, vB_EcoM_EP57, and vB_EcoM_EP69. Only the phage vB_EcoP_EP32b had an increase in phage titre as time progressed. Phage vB_EcoM_EP57 revealed the highest reduction of growth with time, from 220 to 117 pfu/ml, while the growth of phage vB_EcoP_EP32b at 40 °C reduced with time, from 65.67 to 53.33 pfu/ml. Phage growth at 40 °C when incubated for 15 to 60 min is depicted in Fig. 6.

### Effect of pH on *E. coli* O157:H7 phages

**Fig. 7:**
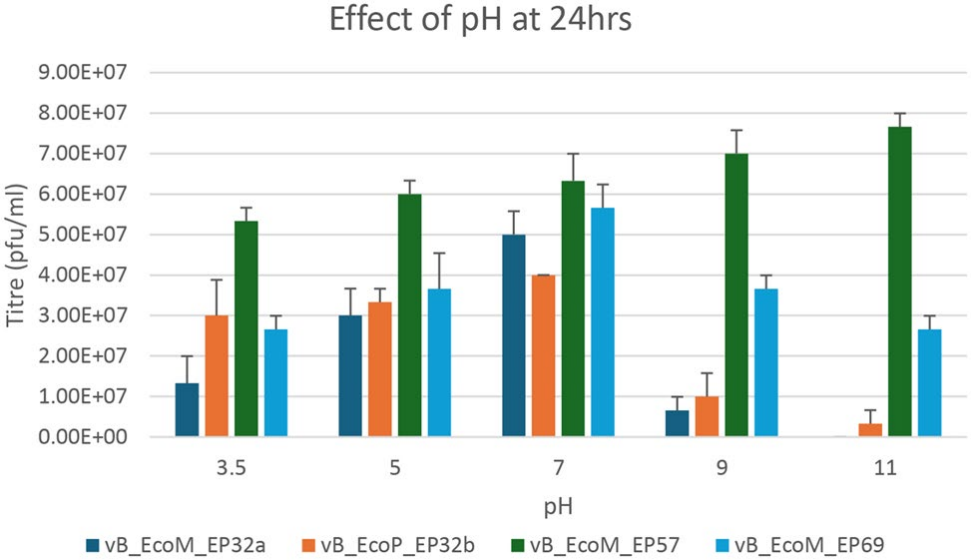
Effect of pH on *E. coli* O157:H7 phage after 24 h

**Fig. 8:**
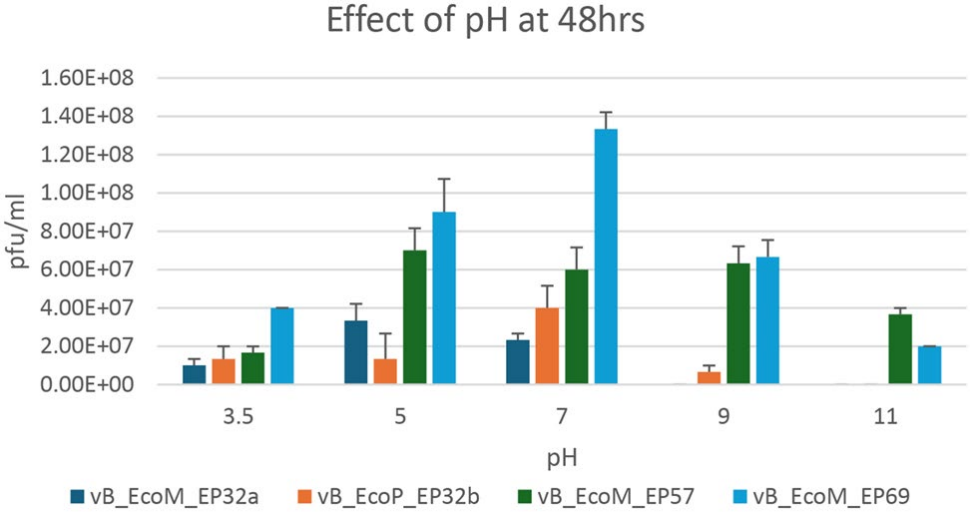
Effect of pH on *E. coli* O157:H7 phage after 48 h

Results from the effect of pH on phage stability when exposed to medium with varying pH differed after 24 and 48 h (Figs. 7 and 8), respectively. When exposed for 24 h, pH 7 was optimal for all phages except phage vB_EcoM_EP57, which had optimum activity at pH 11. However, when exposed for 48 h, the activity of phage vB_EcoM_EP57 was reduced at pH 11, while phage vB_EcoM_EP69 had an increase in activity at pH 7 after 48 h.

### Genomic features of Escherichia phage vB_EcoM_EP32a, vB_EcoP_EP32b and vB_EcoM_EP57

Whole genome analysis on PHASTER and RAST showed the different genomic features of all three phage genomes (Table 4). The genomes were renamed as vB_EcoM_EP32a, vB_EcoP_EP32b, and vB_EcoM_EP57 and deposited into the NCBI and the following ascension numbers were obtained: OR544954, OR544955, and OR544956 for *Escherichia* phages vB_EcoM_EP32a, vB_EcoP_EP32b, and vB_EcoM_EP57, respectively.

**Table 4:** Genomic features of *Escherichia* phage vB_EcoM_EP32a, vB_EcoP_EP32b, and vB_EcoM_EP57 genomes

Features	Escherichia phage vB_EcoM_EP32a	Escherichia phage vB_EcoP_EP32b	Escherichia phage vB_EcoM_EP57
Class	Caudoviricetes	Caudoviricetes	Caudoviricetes
Order	Caudovirales	Caudovirales	Caudovirales
Family	Straboviridae	Straboviridae	Autographiviridae
Genus	*Mosigvirus*	*Mosigvirus*	*Phapecoctavirus*
Nucleic acid	Linear dsDNA	Linear dsDNA	Linear dsDNA
Ascension number	OR544954	OR544955	OR544956
Genome size	163,906 bp	156,698 bp	130,723 bp
GC content (%)	37.61%	37%	39%
Total genes	279	271	305
total number of proteins	277	271	294
Total number of tRNAs	2	0	11
Total number of ORFs	61	68	61

*The Escherichia* phage vB_EcoM_EP32a genome contained 279 genes. A large proportion (277, 99.3%) of the genes were coding for proteins, and only 2 (0.7%) were coding for tRNAs. A proportion of protein-coding genes (149, 54%) were hypothetical proteins, while 128 (46%) were known proteins. The *Escherichia* phage vB_EcoP_EP32b genome contained 271 genes with no tRNA. A proportion of protein-coding genes (130, 48%) were hypothetical proteins, while 141 (52%) were known proteins. The *Escherichia* phage vB_EcoM_EP57 genome contained 305 genes. A large proportion (294, 96.4%) of the genes were coding for proteins, and 11 (3.6%) were coding for tRNA. A large proportion of protein-coding genes (280, 90.2%) were hypothetical proteins, while 14 (9.8%) were known proteins. Table 5 shows the various types of tRNA produced by each phage genome except for vB_EcoP_E32b that does not have any tRNA. *Escherichia* vB_EcoM_EP32a and vB_EcoP_EP57 genomes both contained 61 ORFs, with 53 (86.9%) predicted to encode for functional proteins (putative proteins associated with phages), and 8 (13.1%) ORFs produced no hit on BLASTp. The *Escherichia* phage vB_EcoP_EP32b genome contained 68 ORFs, with 55 (80.8%) predicted to encode for functional proteins (putative proteins associated with phages), and 13 (19.2%) ORFs produced no hit on BLASTp. The functional proteins were distributed into four groups, hypothetical proteins, structural proteins, replication/transcription related genes and lysis related proteins. The distribution of the proteins in the genome are shown in Table 6.

**Table 5:** Types of tRNA found in phage genomes

Phage	tRNA type	Length	tRNA beginning	tRNA End	Anti-codon
vB_EcoM_EP32a	Met	74	108,353	108,426	CAT
	Arg	77	108,433	108,509	TCT
vB_EcoM_EP57	Met	72	87,515	57,444	CAT
	Arg	72	87,438	87,367	TCT
	Ser	83	87,033	86,951	GCT
	Tyr	85	86,869	86,785	GTA
	Asn	83	86,775	86,693	GTT
	Thr	72	86,599	86,528	TGT
	Gly	74	86,208	86,135	TCC
	Gln	73	86,036	85,964	TTG
	Pro	74	85,867	85,794	TGG
	Ile	71	85,784	85,714	GAT
	Met	78	85,631	85,554	CAT

**Table 6:** Distribution of protein types amongst phage ORFs

Protein types	vB_EcoM_EP32a	vB_EcoP_EP32b	vB_EcoM_EP57
Total number of ORFs	61	68	61
No hit	8	13	8
Hypothetical protein	15	10	35
Structural protein	5	21	4
Replication/Transcription related ORFs	28	23	12
Lysis related ORFs	5	1	2

**Fig. 9:**
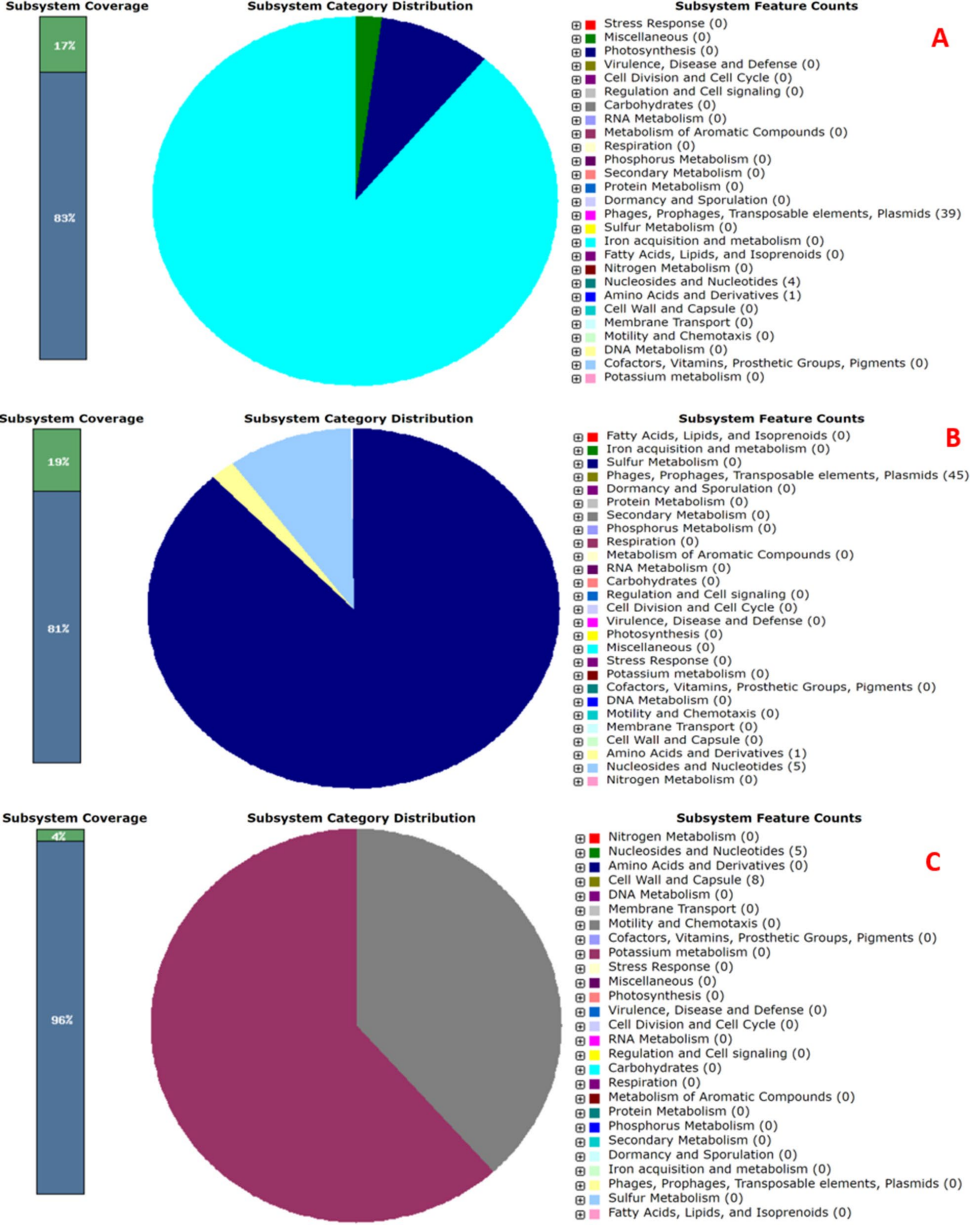
Subsystem function of *Escherichia* phage vB_EcoM_EP32a (**A**), vB_EcoP_EP32b (**B**), and vB_EcoM_EP57 (**C**)

**Fig. 10:**
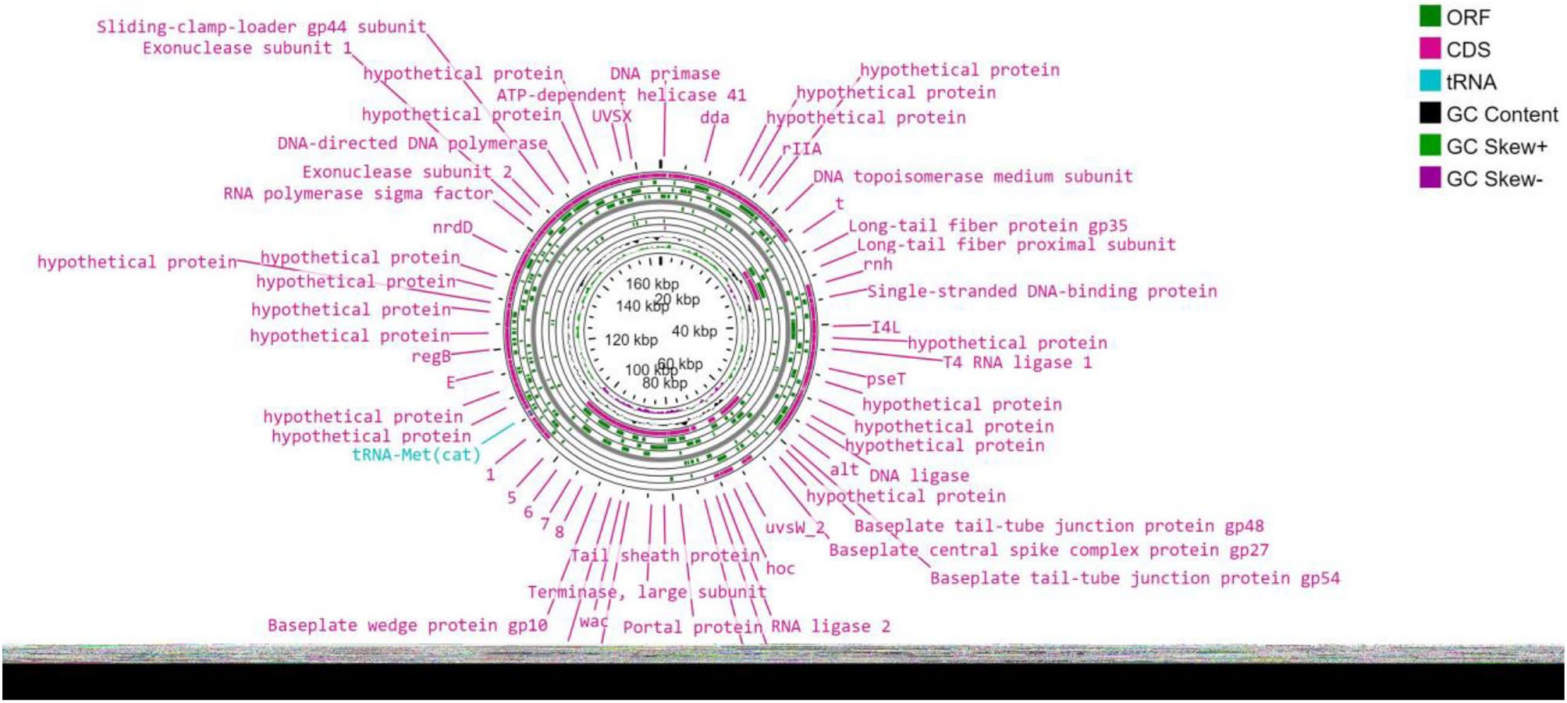
Genomic map of *Escherichia* phage vB_EcoM_EP32a

**Fig. 11:**
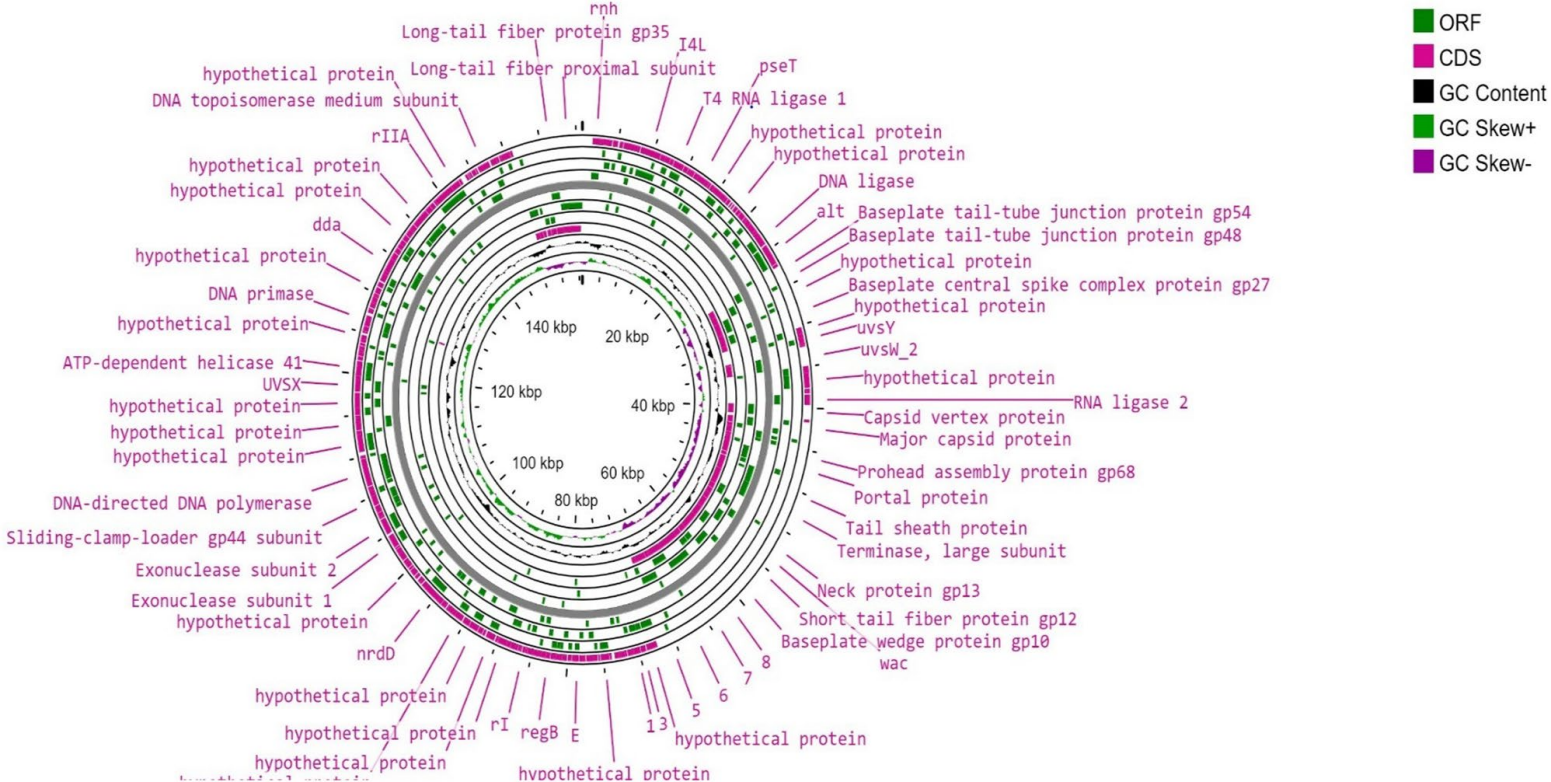
Genomic map of *Escherichia* phage vB_EcoP_EP32b

**Fig. 12:**
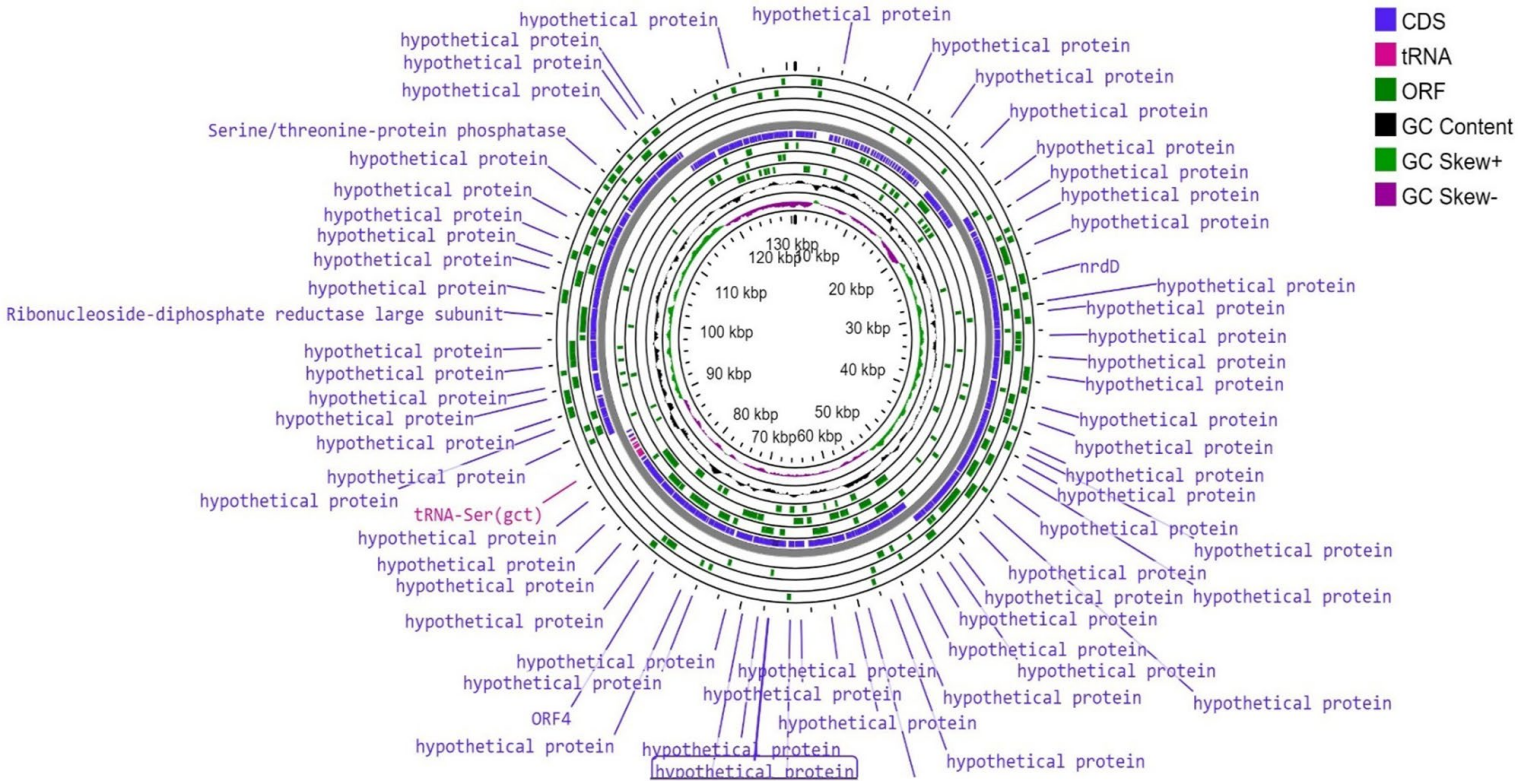
Genomic map of *Escherichia* phage vB_EcoM_EP57

As depicted in Fig. 9 (A, B, and C), the *Escherichia* phage vB_EcoM_EP32a genome contained 39 phage features (phage tail fibre and phage capsid proteins), 1 amino acid and derivatives, and 4 nucleosides and nucleotides. *Escherichia* phage vB_EcoP_EP32b has 45 phage features (phage tail fibre and phage capsid proteins), 1 “amino acid and derivatives” and 5 “nucleosides and nucleotides” while *Escherichia* phage vB_EcoM_EP57 has 8 “cell walls and capsules” and 5 “nucleosides and nucleotides”. The genome map of all three phages is depicted in Figs. 10, 11 and 12.

### Phylogenetic analysis and comparative genomic analysis of Escherichia phages

**Fig. 13:**
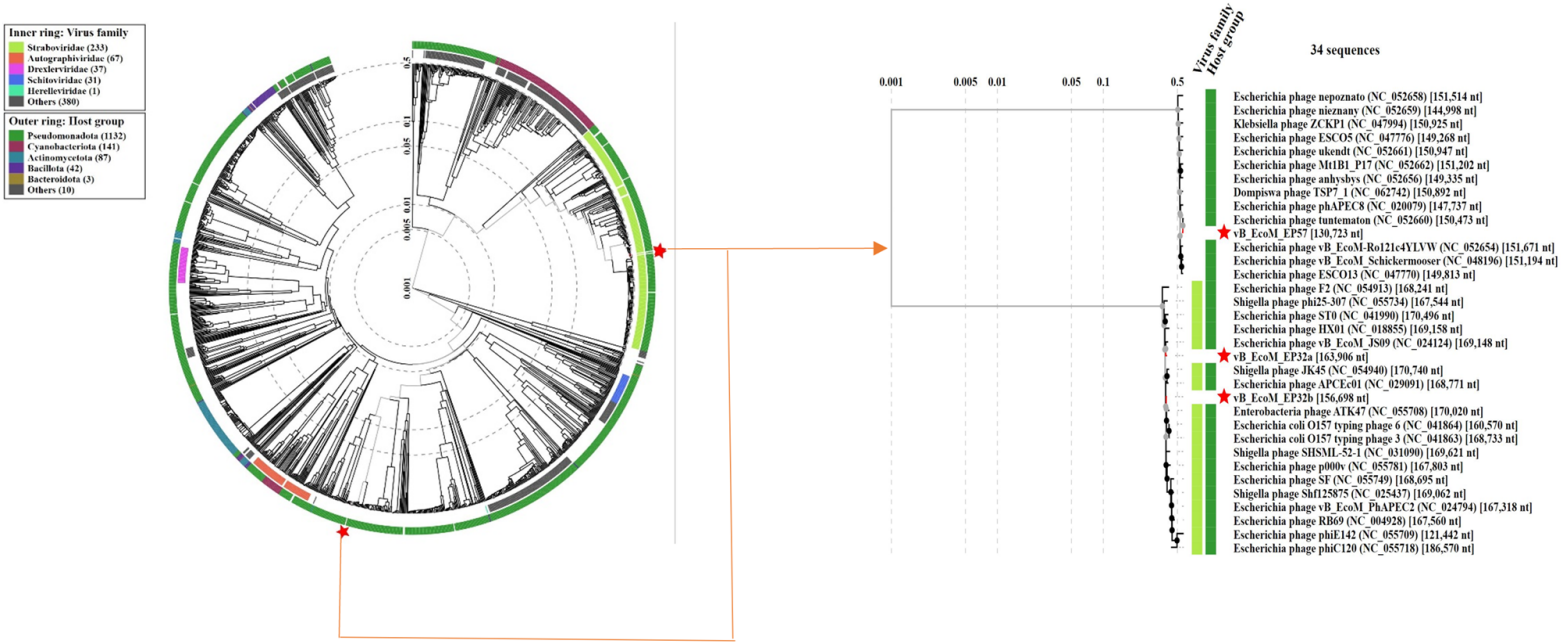
Proteomic tree of *Escherichia* phage vB_EcoM_EP32a, vB_EcoP_EP32b and vB_EcoM_EP57 constructed based on the complete genome sequences of closely related phages selected from the entire proteomic tree on the Viptree software. The tree is constructed by BIONJ based on genomic distance matrixes, and mid-point rooted. Branch lengths are logarithmically scaled from the root of the entire proteomic tree. The numbers at the top represent the log‐ scaled branch lengths based on the SG (normalized tBLASTx scores) values

**Fig. 14:**
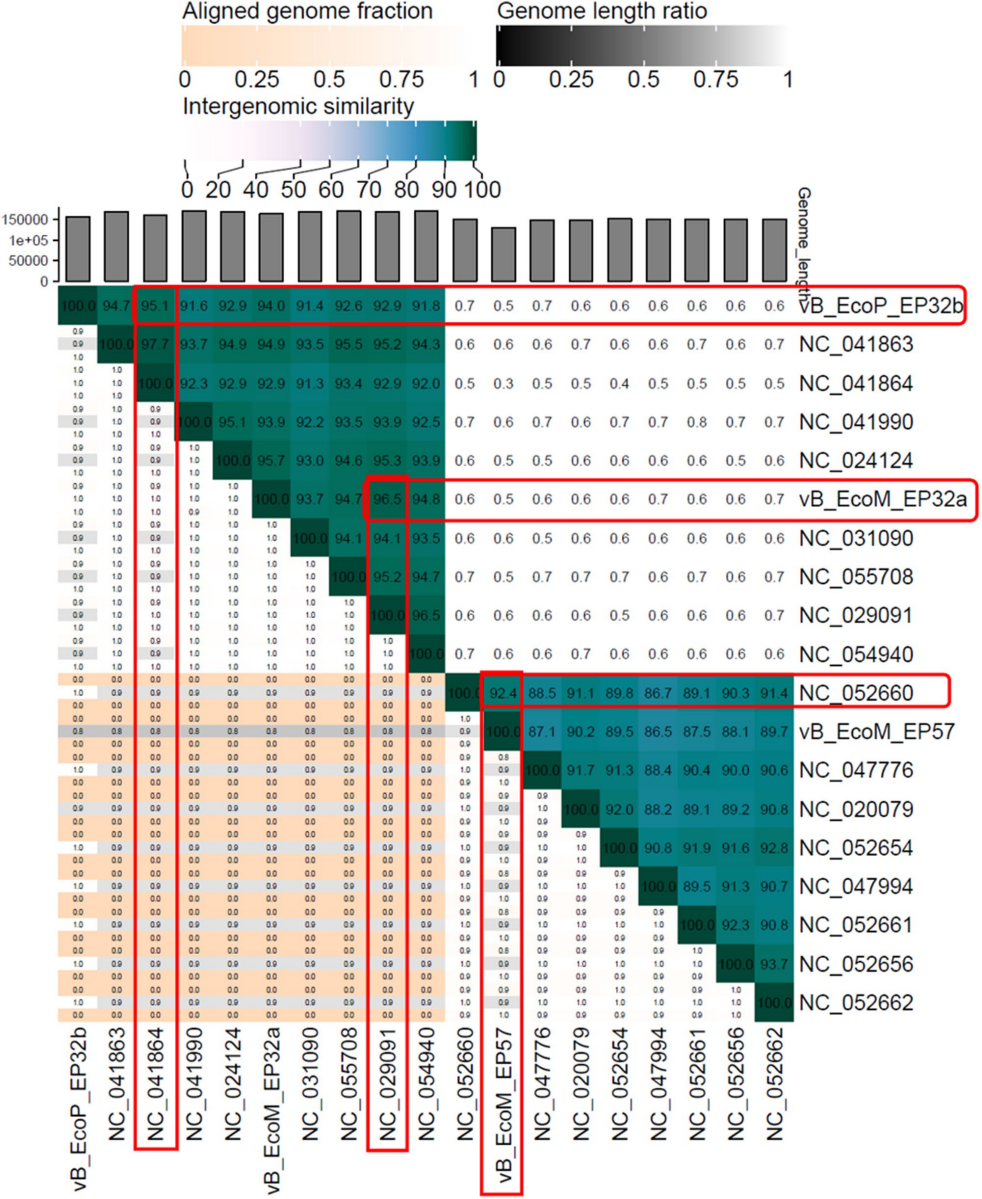
Hit Map for genomic comparison of *Escherichia* phage vB_EcoM_EP32a, vB_EcoP_EP32b, and vB_EcoM_EP57

**Fig. 15:**
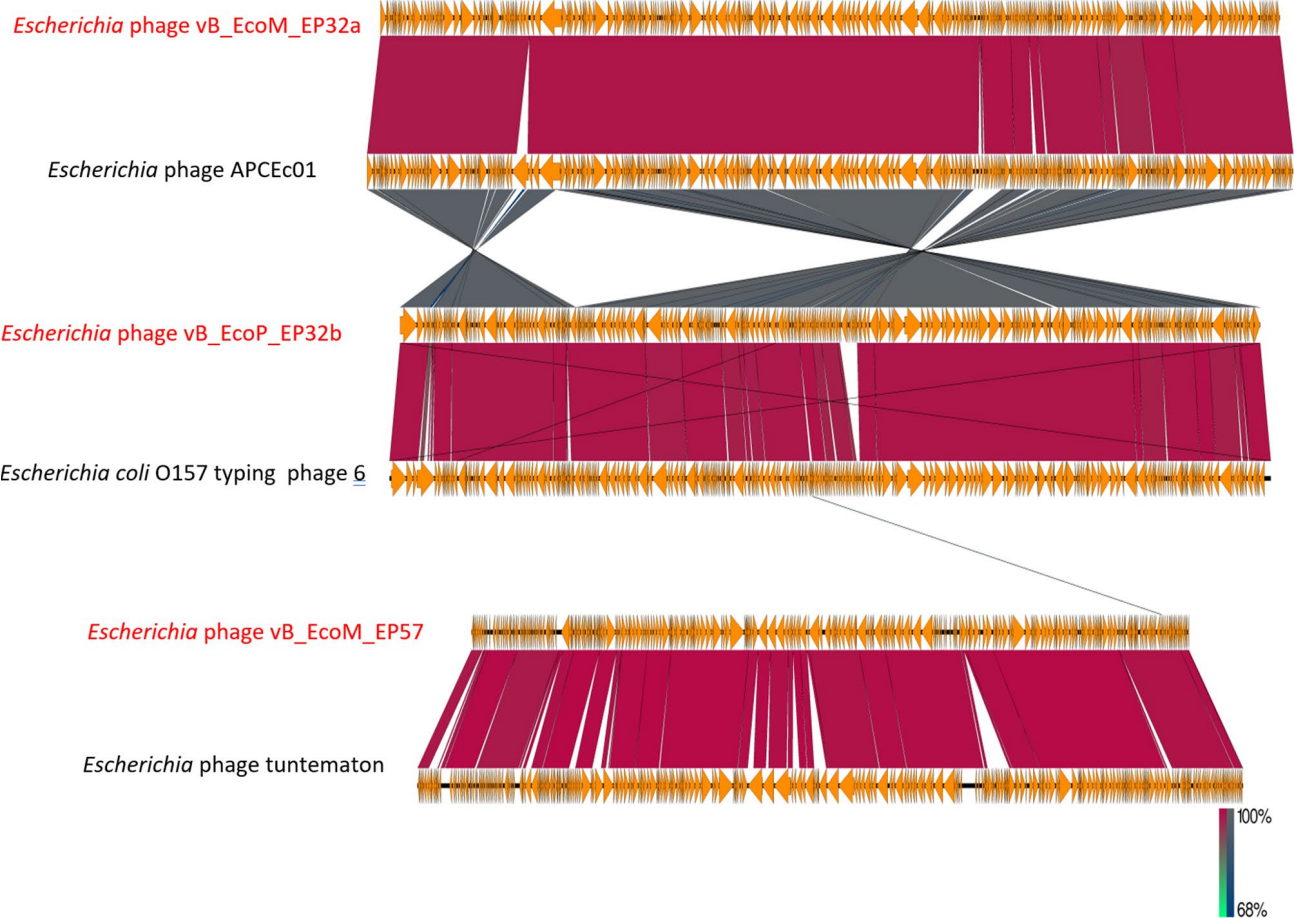
Easyfig homology diagram of vB_EcoM_EP32a, vB_EcoP_EP32b and vB_EcoM_EP57 (with red colour) with close phage relatives in NCBI using BLASTn. Arrows represent the locations of coding sequences and the percentage of sequence similarity is indicated by the red and grey shaded lines

Phylogenetic analysis of phage proteomes using ViPTree revealed that all three phages belong to the host group *pseudomonadota* as shown in the phylogenetic tree in Fig. 13. The VIRIDIC showed the inter-genomic similarities between phages from this study and other closely related phages from the NCBI database (Fig. 14). *Escherichia* phage vB_EcoM_EP32a demonstrated 96.5% intergenomic similarities for *Escherichia* phage APCEc01, *Escherichia* phage vB_EcoP_EP32b demonstrated 95.1% intergenomic similarities for *Escherichia coli* O157 typing phage 6 while *Escherichia* phage vB_EcoM_EP57 demonstrated 92.4% intergenomic similarities for *Escherichia* phage tuntematon. A diagram from the EasyFig software (Fig. 15) showed the comparative analysis of all three phage genomes with their close homologs (*Escherichia* phage APCEc01, *Escherichia coli* O157 typing phage 6, and *Escherichia* phage tuntematon) in BLASTn.

**Fig. 16:**
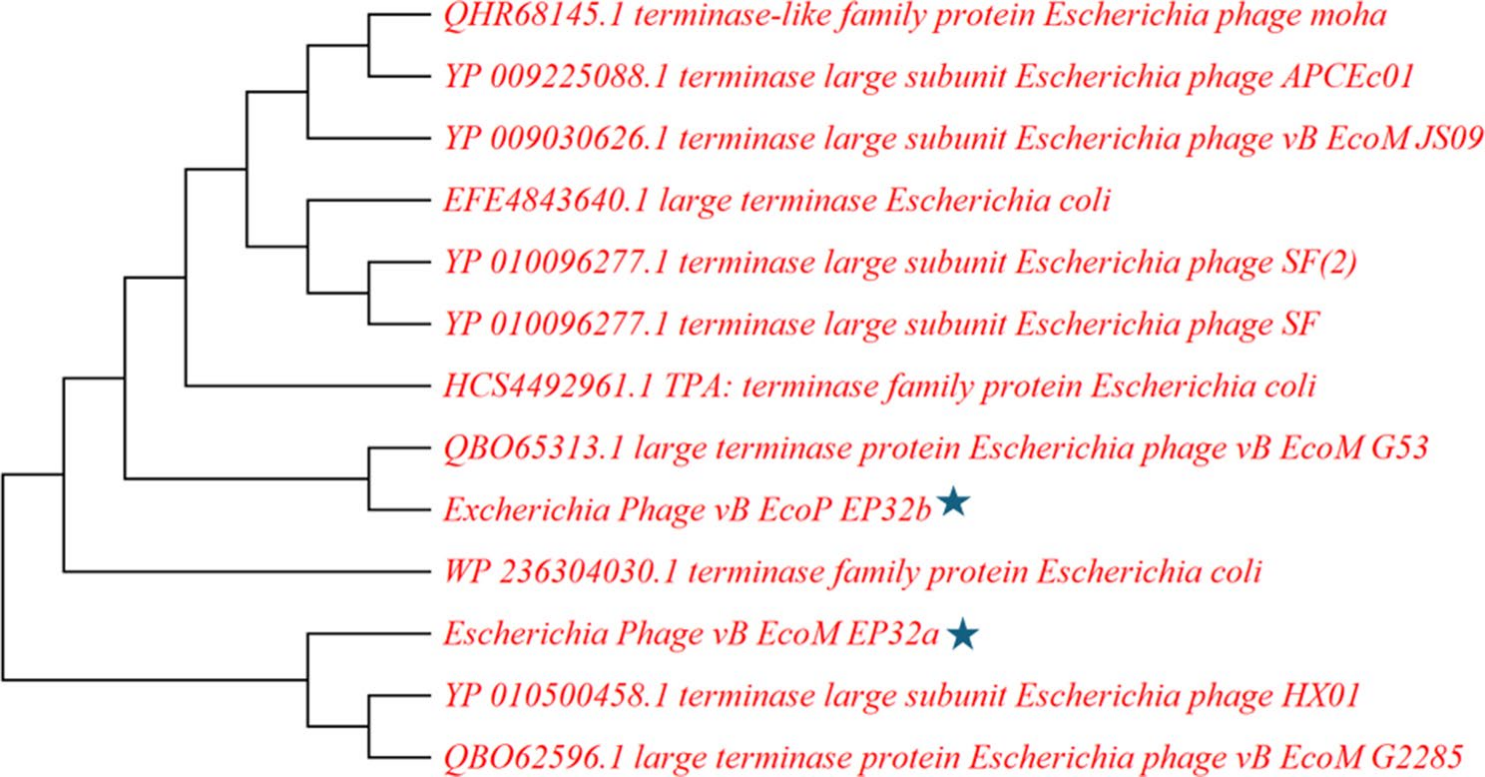
Phylogenetic tree showing position of the *Escherichia* phage vB_EcoM_EP32a and vB_EcoP_32b phage (blue star) based on the amino acid sequence of the terminase large subunit (TerL) of the closest 10 sequences identified using NCBI BLASTp. Sequences were aligned using MUSCLE in MEGA 11 software. The neighbor joining method was used to generate phylogenetic tree

**Fig. 17:**
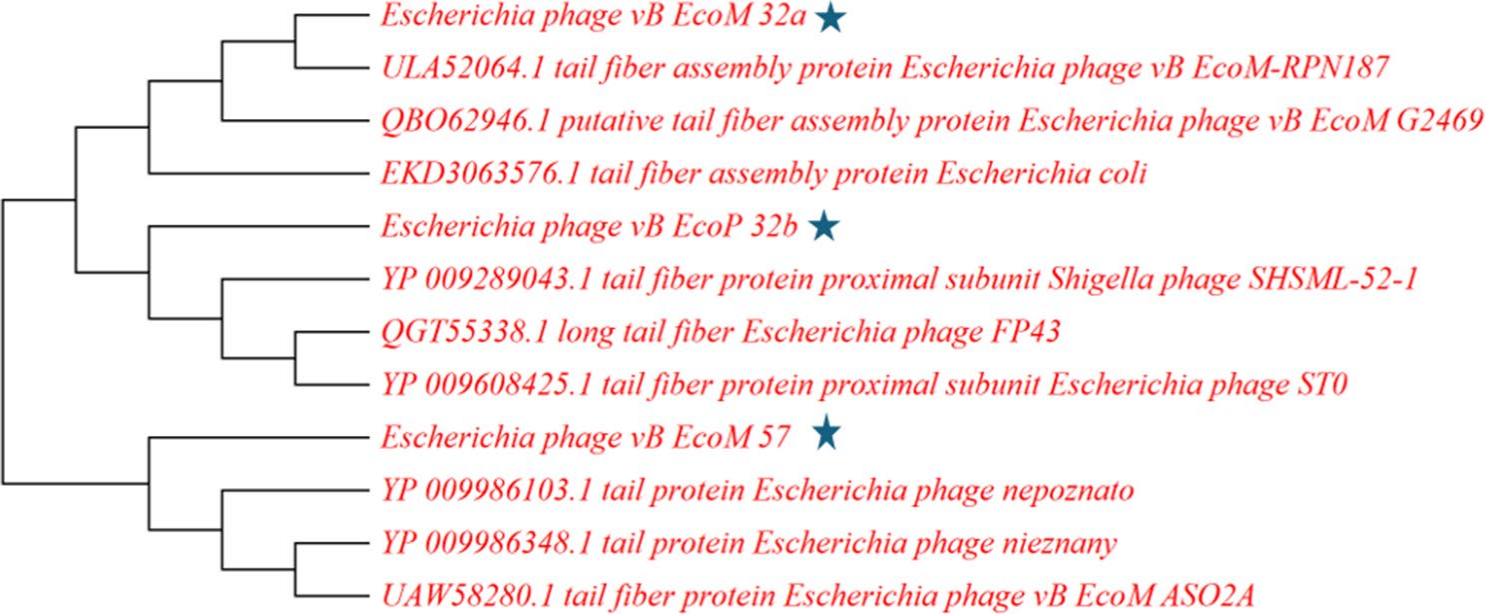
Phylogenetic tree showing position of the phages vB_EcoM_EP32a and vB_EcoP_EP32b and vB_EcoM_EP57 (blue star) based on the amino acid sequence of the tail protein of the closest 9 sequences identified using NCBI BLASTp. Sequences were aligned using MUSCLE in MEGA 11 software. The neighbor joining method was used to generate phylogenetic tree

Two phylogenetic trees were generated using the terminase large subunit (TerL) and tail protein (Figs. 16 and 17). Results showed that *Escherichia* phage vB_EcoM_G53 has the most similar TerL with vB_EcoP_EP32b. Also, *Escherichia* phage HX01 and *Escherichia* phage vB_EcoM_G2285 had similar TerL with vB_EcoM_EP32a. There was no TerL found in vB_EcoM_EP57.

### Safety potential of the Escherichia phages

For the suitability of *Escherichia* phage vB_EcoM_EP32a, vB_EcoP_EP32b, and vB_EcoM_EP57 as probable candidates for therapy or control of bacteria, results obtained from VirulenceFinder, ResFinder, RAST, and Patric annotation showed that all three *Escherichia* phage genomes did not contain toxin proteins, elements associated with lysogeny (integrase), virulence (such as *stx)*, or antimicrobial genes or sequences. Confirmation on PhageLeads showed that there were no genes related to temperate lifestyle, virulence, or antimicrobial resistance.

## Discussion

*E. coli* O157:H7 is the major cause of food-borne infections in humans worldwide [28], and has been classified as a pathogen of severe public health concern, especially because of the rising rate of antibiotic resistance amongst this serotype. The rapid rise of antibiotic-resistant bacteria has influenced the use of phages as an alternative strategy to combat multi drug resistant pathogens [29, 30] which has yielded encouraging results [31, 32].

Lytic phages are preferred over lysogenic phages for phage therapy and biocontrol applications due to their ability to totally lyse bacteria and limit horizontal gene transfer between bacterial populations [33]. This is shown by the presence of well-defined zone of lysis against the pathogen as produced in this study. Phages had plaque sizes varying from 1 mm to 2 mm and phage titers that ranged from 13 × 10^5^ to 35 × 10^9^ PFU/mL, which is similar to *E. coli* phage isolated from a previous study [34].

The lytic spectrum of a phage is a crucial trait that determines its ability to eliminate specific bacterial genera, species, and strains [35]. This simply means that phage candidates for biocontrol must possess a wide host range [36]. In this study, *E. coli* O157:H7 phages exhibited a broad lytic spectrum, as clear plaques were observed on other environmental *E. coli* isolates, *S. aureus*, and *P. aeruginosa (*ATCC 27853).

Following phage isolation, classifying phages into taxonomic groups is an essential step. The TEM technique is straightforward and dependable when evaluating the structural properties, taxonomic categorization, and morphological characterization of bacteriophages [37]. All four phages are tailed phages, hence belonging to the class *Caudoviricetes.* Prior research has indicated phages belonging to *Caudoviricetes* exhibit the greatest diversity, prevalence, and distribution among all bacterial viruses [38].

Phage candidates for biocontrol applications should have a short latent period and a large burst size [39], simply because they are more successful in inactivating bacteria [40]. The current study showed that, phages vB_EcoM_EP32a, vB_EcoP_EP32b, and vB_EcoM_EP69 had a latent period of 15 min, whereas phage vB_EcoM_EP57 had the longest latent period of 20 min. Phage vB_EcoP_EP32b had the smallest burst size (200 PFU/ml), while phages vB_EcoM_EP32a and vB_EcoM_EP69 had relatively high burst sizes of 392 and 360 PFUs, respectively. These findings support the findings of Manohar, Tamhankar [14] who reported *Escherichia* phage myPSH1131 having latency period of 20 min but had a lower burst size of 130 phage particles/infected cells. The fast infection and multiplication of these phages, however, suggests that they could produce enough virions within a short period of time to lyse the host bacteria, making them desirable candidates for a biocontrol treatment program.

Adsorption is the attachment or binding of phages to the surface of their host bacterial cells [41]. Quantifying phage adsorption rates is essential for designing phage-based treatments for bacterial illnesses and understanding phage-host interactions [42]. Also, quantifying adsorption rates can help in identifying phage-resistant bacteria through the measurement of free (unadsorbed) phages after incubation. In this study, more than 50% (67.22 ± 5.5%) of all the four phages were adsorbed to the bacteria after 1 min incubation period. A high adsorption rate (> 90%) was recorded after 15 min. These findings were consistent with previous study that reported an adsorption rate of 99.5% after 15 min [43]. The ability of the phages to attach to the bacteria quickly can lead to more rapid therapeutic action which can be particularly beneficial in severe or life-threatening infections where a quick reduction in the bacterial load is needed.

Several factors, such as temperature and pH, can affect the stability and viability of phages [44]. As a biocontrol agent in the food industry, phage candidates should have a long shelf life and be viable at distinct temperatures such as storage temperature (4 °C), room temperature (28 °C), and body/incubating temperature (37 °C) [45]. These temperatures were selected for incubation and phages in this study were viable at all three temperatures making them suitable for use in the food industry. Furthermore, the digestive system of cattle has a temperature range of 37 to 40 °C. The fact that phages can endure these temperatures implies that they can be used as biocontrol agents on living animals. Phages were also viable at all tested pH (3.5, 5, 7, 9, and 11) levels after 24 h, however after 48 h, phages vB_EcoM_EP32a and vB_EcoP_EP32b reduced in activity at pH 11. This finding corroborates the report of Yuan, Zhang [27] who observed a strong tolerance of phage isolate between pH 5 to 11. Considering the stability of the phages at pH 5 and 7, they can consequently be applied as a therapeutic agent in cattle as the pH of the rumen ranges between 6 and 7.

Genome annotation of studied *E. coli* O157 phages showed genome sizes between 130, 000 bp and 165,000 bp; this supports the studies of Cowley, Beckett [46], which reported *E. coli* O157 phages with gene sizes > 130,000 bp. In this study, GC contents of 37.61, 37.55, and 39% were obtained for *Escherichia* phage vB_EcoM_EP32a, vB_EcoP_EP32b, and vB_EcoM_EP57, respectively. The study of Tang, Tang [47] also reported the GC content of *E. coli* phage to be 35.4%.

All three phage genomes possessed linear dsDNA. While the *Escherichia* phage vB_EcoM_EP32a had a percentage indent of 98.08% to reference phage *Escherichia* phage APCEc01, phage vB_EcoP_EP32b had a percentage indent of 97.87% to *Escherichia coli* O157 typing phage 6. Both reference phages belong to the family *Straboviridae*. *Escherichia* phage vB_EcoM_EP57 was similar to *Escherichia coli* tuntematon, with a percentage identity of 99.37%, which belongs to the *Autographiviridae* family. All *Escherichia* phage genomes contained features similar to those of their respective reference strains. Based on these similarities, the *Escherichia* phages vB_EcoM_EP32a and vB_EcoP_EP32b were classified under the class *Caudoviricetes* and *family Straboviridae*, while the *Escherichia* phage vB_EcoM_EP57 was classified under the class *Caudoviricetes* and family *Autographiviridae*. Also, *Caudoviricetes* are a class of phages that possess a tail [48]. Hence, these findings are consistent with the TEM results obtained.

While most phages only have one or two tRNA genes, others have more than 20 of these genetic sequences [49]. A recent study reported 19 tRNA genes within the genome of an *Escherichia* phage [50]. Although phages predominantly rely on the molecular machinery of the host cell for replication, the presence of tRNA genes in the phage genome facilitates protein synthesis [51]. Two of the *Escherichia* phages (vB_EcoM_EP32a and vB_EcoM_EP57) characterised in this study had proteins coding for tRNA. Phages with tRNA in their genomes have been associated with strong lytic activity, as well as a distinct preference for codons that differ from those chosen by their hosts [52]. This supports the possible biocontrol applications of the *Escherichia* phages characterised in this study.

Known proteins observed in phage genomes in this study include DNA helicase, exonuclease, transcriptional regulator, tail fibre, lysozyme, and holin. The presence of these proteins in the phage genome ensures the successful replication and propagation of phages within the host bacterium [53, 54]. Furthermore, their presence in *Escherichia* phages vB_EcoM_EP32a and vB_EcoP_EP32b are highly significant in phage therapy as they aid in lysis, efficient phage replication and overall safety of phage-based treatment hence, making these phages suitable candidate for phage therapy. *Escherichia* vB_EcoM_EP32a and vB_EcoM_EP57 genomes both contained 61 ORFs, with 53 (86.9%) predicted to encode for functional proteins, which are putative proteins associated with phages, and 8 (13.1%) ORFs of both genomes produced no hit on BLASTp. *Escherichia* phage vB_EcoM_EP32a had 5 ORFs coding for structural protein, 28 responsible for DNA replication/transcription and 5 associated with lysis. *Escherichia* vB_EcoM_EP57 had 4 ORFs coding for structural protein, 12 responsible for DNA replication/transcription and 2 associated with lysis. The phage vB_EcoP_EP32b genome contained 68 ORFs, with 55 (80.8%) predicted to encode for functional proteins (putative proteins associated with phages). The genome had 21 ORFs coding for structural protein, 23 responsible for DNA replication/transcription and 1 associated with lysis. Hits on BLASTp showed 15 hypothetical proteins for vB_EcoM_EP32a, 10 hypothetical proteins for vB_EcoP_EP32b, and 35 hypothetical proteins for vB_EcoM_EP57. Proteins with unknown functions have been frequently observed in phage genomes [55, 56]; however, the large number of hypothetical proteins in vB_EcoM_EP57 makes it difficult to fully characterize the phage genome.

Findings from the current study showed that phage vB_EcoM_EP32a and vB_EcoP_EP32b are closely related to each other. This may be attributed to the fact that they were isolated using the same bacteria host (*E. coli* O157:H7 -J32). The inter-genomic similarities between phages from this study and other closely related phages revealed high percentage similarities on VIRIDIC. The inter-genomic similarity between *Escherichia* phage vB_EcoM_EP32a and *Escherichia* phage APCEc01 was determined to be 96.5%, vB_EcoP_EP32b to *Escherichia coli* O157 typing phage 6, to 92.4%, and vB_EcoM_EP57 to *Escherichia* phage tuntematon to 92.4%.

Genomic comparison using the terminase large subunit (TerL) and terminase small subunit (TerS) proteins showed that *Escherichia* phage vB_EcoM_G53 has the most similar TerL to VB_EcoP_EP32b. Also, *Escherichia* phage HX01 and *Escherichia* phage vB_EcoM_G2285 had the most similar TerL to vB_EcoM_EP32a. While *Escherichia* phage vB_EcoM_G53 and *Escherichia* phage vB_EcoM_G2285 were isolated from manure in Germany [57], *Escherichia* phage HX01 was isolated from Duck faeces in China [58]. There was no TerL found in vB_EcoM_EP57. Furthermore, based on their tail protein, vB_EcoM_EP32a is closely related to *Escherichia* phage vB_EcoM-RPN187, and vB_EcoP_EP32b is closely related to *Shigella* phage SHSML-52-1. Both reference phages belong to the family *Straboviridae*, which further confirms the phylogenetic classification of *Escherichia* phages vB_EcoM_EP32a and vB_EcoP_EP32b. Phage vB_EcoM_EP57 on the other hand is closely related to *Escherichia* phage nepoznato which also belongs to the class *Caudoviricetes*.

In terms of phage safety for potential use as a biocontrol, a key feature is that the phage candidate must be exclusively lytic, and the genome should lack the integrase enzyme, virulence genes, and antimicrobial resistance markers [25]. Transduction-mediated horizontal gene transfer may facilitate the acquisition and transfer of virulence and multi drug resistance genes to other bacterial pathogens [59], and so assessing the phage genome for the presence of these genes is crucial before its use as a biocontrol agent. Current findings showed that all three *Escherichia* phage genomes did not contain toxin proteins, elements associated with lysogeny (integrase), virulence (such as *stx)*, or antimicrobial genes or sequences; hence, may be regarded as safe for biocontrol purposes.

## Conclusion

This study emphasizes the need to assess the safety of phages for their prospective use as a biocontrol. The study aimed to thoroughly investigate the genomic makeup of *E. coli* O157:H7 phages, recognising the increasing concern about antibiotic-resistant pathogens and the prospective role phages can play in managing this issue. The study provided substantial details on the safety of these phages with the use of WGS. Assessing the genetic composition revealed that phages in this study lacked unfavorable genes related to lysogeny, antibiotic resistance, and virulence. This not only confirmed their appropriateness for biocontrol but also demonstrated the possibility of their safe usage in the food sector and clinical trials. The study also acknowledged the impact of recent advances in sequencing technologies and bioinformatic tools on improving phage taxonomy and safety evaluation. These advancements have resulted in a more precise classification system based on phage genome similarities and relatedness, thereby allowing for the identification of phages. As the field of phage therapy and biocontrol evolves, the findings described in this study contribute to the ongoing efforts to prevent bacterial infection and improve food safety.

## Data Availability

The datasets generated and/or analysed during the current study are available in the NCBI repository, [Accession Numbers: OR544954, OR544955, and OR544956].

## Abbreviations

AMR: Antimicrobial resistance
DNA: Deoxyribonucleic acid
PGAP: Prokaryotic genome annotation pipeline
RAST: Rapid annotation using the subsystem technology
RGI: Resistance gene identifier
TEM: Transmission electron microscopy
WGS: Whole genome sequencing
SDS: Sodium dodecyl sulphate
PEG: Polyethylene glycol
CGE: Centre for Genomic Epidemiology
ORF: Open reading frame
ICTV: International Committee on Taxonomy of Viruses
PATRIC: Pathosystems Resource Integration Centre
PHAST: Phage Search Tool
